# Exploring end-to-end earthquake early warning performance in large earthquakes using the February 2023 Kahramanmaraş, Türkiye sequence

**DOI:** 10.1038/s41598-025-29755-z

**Published:** 2025-12-12

**Authors:** Savvas Marcou, Angela I. Lux, Andrei Akimov, Amy L. Williamson, Richard M. Allen

**Affiliations:** 1 Berkeley Seismological Laboratory, University of California, Berkeley, 94703 Berkeley, CA USA; 2 Department of Earth and Planetary Science, University of California, Berkeley, 94703 Berkeley, CA USA

**Keywords:** Earthquake, Earthquake early warning, Simulation, Natural hazards, Public alerting, Natural hazards, Solid Earth sciences

## Abstract

Earthquake early warning systems (EEWS) aim to warn end-users of impending ground shaking. They can be most impactful in large earthquakes occurring close to large population centers, where exposure to strong ground shaking is extensive. However, such earthquakes are rare, and EEWS performance expectations remain uncertain. The February 2023 Kahramanmaraş, Türkiye sequence, including the M7.8 Pazarcık and M7.5 Elbistan events, exposed millions to strong ground shaking and produced a rich waveform dataset, offering a test case. We use this data to produce a realistic simulation of warning times. We use the EPIC point source algorithm for real-time earthquake characterization, and incorporate alert delivery latency using a statistical model driven by real-world alert delivery data from California, collected by the MyShake smartphone app. We show EPIC would produce solutions very quickly (4 s for Pazarcık, 10 s for Elbistan). Despite EPIC’s expected magnitude underestimation (peak M6.7 for Pazarcık, M7.2 for Elbistan), we show that its magnitude estimate grows large quickly enough to provide areas of MMI 6+ shaking with up to 20 s of warning time, even with alert delivery latencies included, provided that low alerting thresholds of MMI 3 or 4 are used.

## Introduction

Earthquake early warning systems (EEWS) aim to alert users of impending strong shaking before it reaches their location^1^. Using dense, distributed sensor networks, EEWS can rapidly detect and characterize earthquakes in real-time, and using fast delivery technologies, alerts are delivered faster than the propagation of strong ground shaking. EEWS alerting is growing worldwide, thanks to both seismic network-based and low-cost sensor systems (e.g., based on smartphones)^1–3^. EEWS provide the greatest benefits in densely populated areas during large earthquakes, which, though rare, cause extensive damaging shaking^4–6^. The largest magnitude events to test national EEWS in real-time have been offshore subduction zone events, with the M9.1 Tohoku, Japan earthquake remaining the largest to date^7,8^. While large (M6.5+) onshore events have tested Taiwan’s EEWS (e.g., the M7.4 April 2024 Hualien earthquake ^9^), very large (M7.5+) onshore ruptures directly beneath large population centers - where system response times are minimal - have not tested traditional network-based EEWS. Consequently, their end-to-end performance, from real-time event characterization to alert delivery, remains uncertain. California, for example, has this type of exposure profile and access to the ShakeAlert EEWS^10,11^, but has not experienced such earthquakes in the modern era.

EEWS function as pipelines with two main distinct elements: alert issuance (real-time event characterization and issuance of ground-motion forecasts) and alert delivery. The speed and accuracy of these distinct elements determine warning times. EEWS use real-time seismic and/or geodetic sensors to rapidly detect ongoing earthquakes and estimate peak ground shaking distribution and intensities. This involves either estimating point (e.g., EPIC^12^) or finite source (e.g., FinDer^13^) parameters and forecasting ground shaking via ground-motion models, or by forward-predicting shaking using shaking already observed (e.g., PLUM^14,15^). End users (e.g., the public, automated systems) are alerted if estimated shaking at their location exceeds a critical alerting threshold. Lowering this threshold tends to ensure more timely alerts^16–19^.

Alerts, once issued, must be delivered to the public, a task often handled by systems distinct to alert issuance systems. Such delivery systems include smartphone apps and TV/radio announcements^1^. These systems often introduce additional delays (alert delivery latencies), but these are often ignored in EEWS warning time performance^13,16,20^. However, recent analyses of public alert delivery latencies for the US-based MyShake smartphone platform^21,22^ and the Wireless Emergency Alerts system^23^ reveal significant delays (on the order of a few seconds). As such, analyses of EEWS performance must jointly consider alert issuance times, alerting thresholds, and alert delivery latency.

**Fig. 1:**
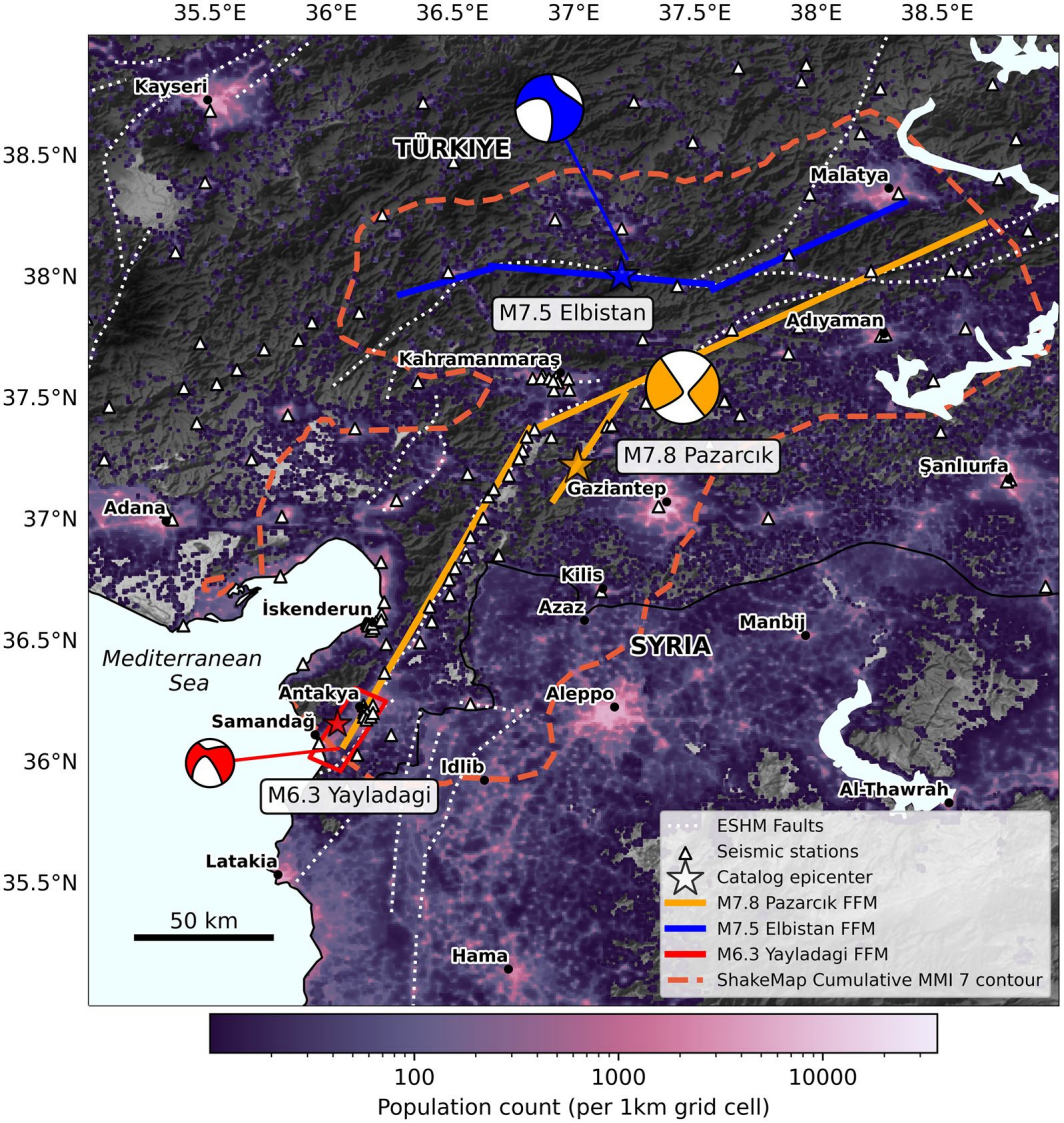
Overview map of the affected area. Map shows population counts in the area from the WorldPop database^39^, at 1 km resolution. White dotted lines show active faults from the European Seismic Hazard Model (ESHM)^40,41^. Locations of seismic stations are shown as white triangles. We plot the epicentral locations and centroid moment tensors for the M7.8 Pazarcık, M7.5 Elbistan, and M6.3 Yayladagi events. Epicenters are taken from the USGS Comprehensive Catalog (ComCat), while moment tensors are taken from the Global Centroid Moment Tensor catalog^42,43^. We plot fault planes for the Pazarcık and Elbistan events from the US Geological Survey (USGS) finite fault models, and for the Yayladagi event from Yolsal-Çevikbilen^44^. The orange dashed line shows the cumulative area experiencing strong (MMI VII) or greater shaking, from USGS ShakeMaps.

In this context, the 2023, Kahramanmaraş, Türkiye sequence - comprising a mainshock doublet in a densely populated area - offers an opportunity to evaluate EEWS and set performance expectations. The sequence was recorded in unprecedented detail by the dense Turkish National Strong Motion Network^24^ (see Fig. 1). The first mainshock, the $M_W\,7.8$ Pazarcık earthquake, occurred on February 6, at 3:17 am local time^25^. It initiated on the Sakçagöz-Narlı segment of the Dead Sea Fault, and then bilaterally propagated on the left-lateral, northeast-southwest-trending East Anatolian Fault (EAF), rupturing a total fault length of >300 km^25–27^. The $M_W\,7.5$ Elbistan earthquake followed just over 9 hours after the Pazarcık event, rupturing the east-west-trending Çardak–Sürgü fault^26,28,29^. Dense waveform observations reveal that the two mainshocks caused extremely strong ground shaking^30,31^. Peak intensities reached Mercalli Modified Intensity (MMI) 8-9^25,28^, with the US Geological Survey’s (USGS) ShakeMap products estimating over 10 million people exposed to MMI 7+ (very strong) shaking across the two events (Fig. 1). The ruptures were complex, featuring multiple sub-events^27,29^. Finite fault models (FFMs) show the first-event involved a multi-fault rupture, with a relatively slow early moment release^26,27,29^, while FFMs of the second event suggest a partial supershear rupture^26,29^. Such complex events challenge EEW source characterization algorithms tracking evolving moment release to solve for magnitude in real time. The sequence also involved numerous aftershocks, including the February 20 $M_W\,6.3$ Yayladagi event (the second largest aftershock after a $M_W\,6.6$ event on February 6). The Yayladagi event occurred at the Pazarcık rupture’s southwestern terminus, near the edge of the seismic network.

In this work, we leverage the rich waveform dataset provided by the Kahramanmaraş sequence and recent analyses of public alert delivery latencies^21,22^ to evaluate the potential end-to-end of EEWS and factors influencing on available warning times. We first replay waveforms through the EPIC EEW algorithm to assess its ability to rapidly determine source parameters for the $M\,7.8$ Pazarcık and $M\,7.5$ Elbistan mainshocks and the edge-of-network $M\,6.3$ Yayladagi event.

EPIC is a point-source algorithm used in the US ShakeAlert system^10,11,19,32^ and elsewhere^33,34^. It is known for producing source parameters very quickly^19,35^, but can struggle to locate out- and edge-of-network events^36^ and is expected to saturate its magnitude estimate in large ($M\ge 7$) events^12,32,35^. We use the Kahramanmaraş doublet to verify these observations and test EPIC’s limits in terms of the users who could be alerted when EPIC is acting alone, without support from finite fault algorithms. We test EPIC without any modifications from its ShakeAlert production version. We compare our results with replays using the finite fault algorithm FinDer^37^ and a hybrid point-source approach^38^.

We then use EPIC’s time-evolving source parameter estimates to simulate end-to-end warning times for the two mainshocks, under three commonly used alerting thresholds^19,21,35^ (MMI 3, 4, and 5). We incorporate MyShake’s US West Coast operational setup for full realism. We incorporate delivery latencies via a statistical model developed using real-world MyShake alert delivery data (see Methods). Warning time for an individual user ($t_{warn}$) is modeled as:

1 $$\begin{aligned} t_{warn} = t_S - t_{alert} - t_{delivery} \end{aligned}$$

Here, $t_{alert}$ is the alert issuance time, as set by the EPIC source parameter progression (i.e., using an alert area extent that evolves over time to reflect the evolving magnitude estimate) and the alerting threshold. Alert areas are set following ShakeAlert practice^45^ (see Methods). $t_{delivery}$ is the delivery latency and is set by our statistical latency model. Warning times are computed as the time from alert delivery to S-wave arrival ($t_S$).

We discuss EPIC source parameter accuracy and timing in light of known source properties. Then, we present our simulation results through joint warning time and shaking intensity maps, and discuss implications for choosing of mass public alerting thresholds.

## Results

### EPIC replays

**Fig. 2:**
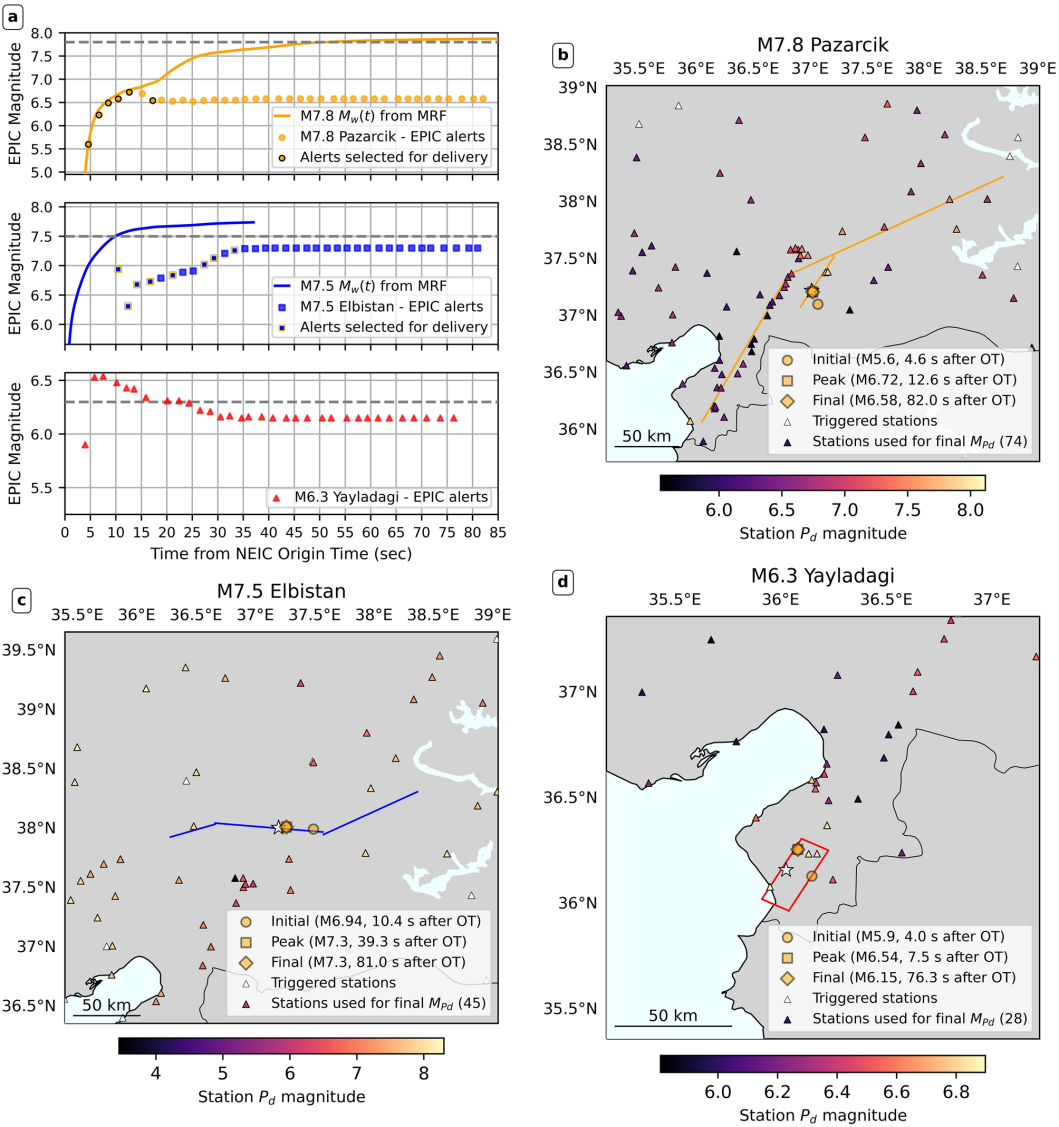
EPIC source parameters for the *M*7.8 Pazarcık, *M*7.5 Elbistan, and *M*6.3 Yayladagi events. (**a**) Evolution of EPIC magnitude estimates for the three replay events. Top panel: Pazarcık; middle panel: Elbistan; bottom panel: Yayladagi. Each EPIC update is shown as a circle. Update timing is calculated with respect to the the USGS National Earthquake Information Center (NEIC) origin time (OT). Updates selected for dissemination in our warning time simulation are outlined. For the Pazarcık and Elbistan events, we also show moment rate functions (MRFs) from Goldberg et al. ^27^, integrated over time into $M_w(t)$ timeseries. The dashed grey line in each panel shows the USGS NEIC catalog magnitude. Note for that the Elbistan event, the catalog magnitude is lower than the magnitude from the integrated MRF; for this event, there is considerable uncertainty in the moment magnitude estimate between different data centers ($M_w\,7.5-7.7$)^27^ (**b**) Evolution of location estimates, and final station magnitudes for the *M*7.8 Pazarcık event. We show the epicenters associated with EPIC’s initial, peak, and final magnitude estimates. White triangles show all stations that were triggered and associated into the EPIC detection of the event, but were excluded from contributing a station magnitude ( $M_{Pd}$), due to being at a distance of $<200\,km$, or failing quality control checks (see Supplementary Text for details). Stations that are colored contributed to the EPIC magnitude estimate, with color indicating the final individual station magnitude. Simplified fault segments are from the FFMs of Goldberg et al. ^27^(Pazarcık and Elbistan events). (**c**) As in (**b**), but for the *M*7.5 Elbistan event. (**d**) As in (**b**), but for the *M*6.3 Yayladagi event. Fault plane (dipping to the NW) from Yolsal-Çevikbilen^44^.

We first analyze the accuracy and timing of real-time location and magnitude estimates from EPIC in our replays of the M7.8 Pazarcık, M7.5 Elbistan, and M6.3 Yayladagi events (Fig. 2). Figure 2 a shows the temporal evolution of EPIC’s magnitude estimates, while panels (b-d) show epicenter estimate evolution and individual station magnitudes derived from peak P-wave displacements (using up to 4 seconds of data post-P-wave trigger). EPIC’s real-time magnitude estimate is a weighted average of individual station magnitudes (see Methods). We compare our replays with Böse et al.^37^, who replayed the same three events using the FinDer finite source algorithm and the same waveform dataset. FinDer provides a best-fit line source estimate by comparing time-evolving peak ground shaking distributions to pre-computed distributions for linear sources^13^. We also compare Pazarcık replays with Rea et al.^38^, who combined a network-based point-source approach with an evolutionary shaking forecast based on single-station data (QuakeUp).

#### M7.8 Pazarcık event

In the simulated real-time replay, EPIC issued its first alert for the Pazarcık event 4.6 s from OT, aided by dense near-epicenter station coverage. This was faster than the first QuakeUp point-source alert (OT+8.8 s). EPIC’s initial location error was 14 km, likely caused by all available triggers being to the north of the epicenter (see Fig. S1). By 7 s from OT, EPIC’s location stabilized with a much improved 1.5 km error (Fig. 2b). EPIC’s initial magnitude estimate was *M*5.6, higher than FinDer’s initial *M*4.4 estimate (at 7 s from OT)^37^. EPIC’s magnitude estimate peaked at *M*6.7 at 12.6 s from OT (see top panel in Fig. 2a), growing in line with the growth of event magnitude shown by the integrated moment-rate function (Fig. 2). In the Pazarcık event, Jia et al. ^29^ resolved a slowly growing initial *M*6.8 subevent on the Narlı fault, which EPIC tracked well using near-fault data. However, the four-second window used to make magnitude estimates meant that subsequent updates missed the rupture’s progression onto the main strand of the EAF. QuakeUp replays^38^ used a longer window and were able to estimate a higher peak magnitude (M7.5 at OT+40 s). FinDer’s magnitude estimate exceeded EPIC’s peak estimate at 18 s from OT^37^. EPIC’s magnitude estimate converged to a final value of *M*6.6 as data from more distant ($\le 200\,km$ from the EPIC epicenter) stations was incorporated.

#### M7.5 Elbistan event

EPIC’s Elbistan event source estimates were less accurate. Much sparser network coverage than the Pazarcık event meant that it took much longer for the minimum of four required triggers to be associated with the event, with the first EPIC alert issued 10.4 s from OT. EPIC’s initial location estimate lay $\sim$26 km east of the catalog epicenter, as three of the four stations used lay east of the true location (see Fig. 2c and Fig. S2) The initial magnitude estimate was *M*6.9. With the correct location, the initial estimate would have been *M*7.0, as contributing stations were roughly equidistant to the true and estimated epicenters. For comparison, FinDer’s initial estimate came 15 s from OT^37^, at *M*5.5. EPIC’s epicenter estimate converged to its final location (5.8 km error) in the second update, 12.4 s from OT, 2 s after the initial alert. EPIC’s magnitude dropped in the second update, but then rose continuously for 25 s (middle panel in Fig. 2a), exceeding *M*7 27 s from OT and stabilizing around *M*7.2, 5 s later. The final *M*7.3 estimate was issued 79 s from OT. FinDer’s magnitude estimate surpassed EPIC’s around 35 s from OT, peaking at *M*7.5.

The snapshot map of individual EPIC station magnitudes (Fig. 2c) shows higher values towards the west of the rupture. This aligns with FFMs of the Elbistan event^26,29^ indicating bilateral rupture with significantly elevated amplitudes and greater rupture velocity towards the west. These observed directivity effects could explain the individual station magnitude pattern (Fig. S4 and Supplementary Text).

#### M6.3 Yayladagi event

Finally, we consider the February, 20, 2023 M6.3 Yayladagi event, which occurred near the Türkiye-Syria border at the edge of the Turkish strong motion network. As expected from previous work^12,32,36^, this edge-of-network event proved challenging for EPIC. EPIC rapidly detected the event (4.0 s from OT), but pushed its initial location 12 km to the east of the catalog epicenter (Fig. 2d). Similar to the Elbistan event, the location was dragged towards the first four stations contributing triggers (Fig. S3). As more stations were incorporated, the location moved to the northeast of the catalog location, with similar error.

In this case, the initial magnitude estimate was *M*5.9 (*M*6.1 if location was correctly estimated). The initial magnitude estimate used stations within 20 km of the event; their close proximity amplified location errors, in line with Williamson et al.^36^. EPIC magnitude rapidly increased to its peak of *M*6.5, 3 s later (7.5 s from OT), a 0.2 unit overestimate (*M*6.7 with the correct location). This was likely caused by S-wave energy in the 4-second window for the three closest stations used by EPIC (Fig. S4). Williamson et al.^46^ identified this issue and proposed revising EPIC’s magnitude scaling to account for S-wave energy at near-field stations. As more station data was incorporated, EPIC’s magnitude estimate gradually reduced, stabilizing at *M*6.2 around OT+30 s. For comparison, FinDer’s initial estimate was *M*5.5 at OT+6 s, peaking at *M*6.6 20 s from OT^37^.

### Developing a MyShake latency model for warning time simulations

**Fig. 3:**
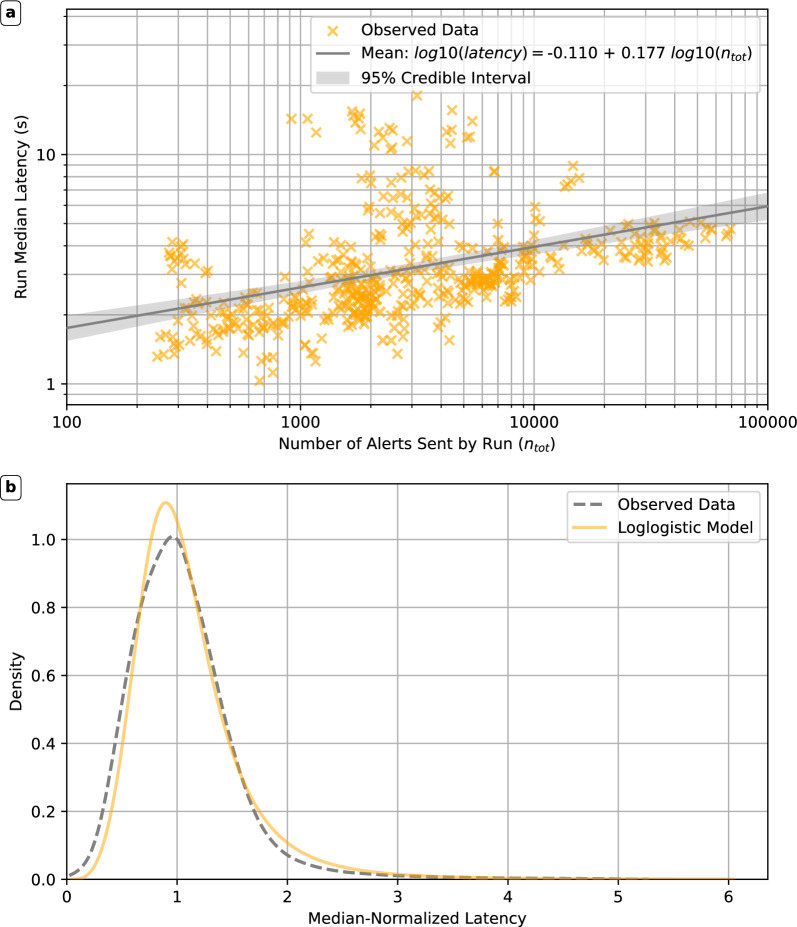
Salient features of MyShake alert delivery latencies. (**a**) Trend of MyShake run median latencies with the number of alerts sent by each run ($n_{tot}$). We also plot our linear model, derived using Bayesian regression, as well as the 95% credible interval for this model. (**b**) Distribution of aggregated MyShake latencies, across all runs and events included in our latency dataset. Dashed line shows a kernel density estimate for the distribution of observed latency. The orange line shows the PDF derived using the mean parameters from the posterior sample for our preferred (loglogistic) latency shape model. Note that we plot the data on a dimensionless scale, with each run’s data divided by the median latency for that run, and then aggregated into a single latency shape dataset.

Realistic, end-to-end warning time simulations in the context of mass public alerting require robust characterization of alert delivery latencies. Alert delivery latencies are generally not reported, but are known to vary across delivery platforms ^18,21–23,47,48^. The MyShake smartphone platform, which has been delivering ShakeAlert EEW alerts to US West Coast users since 2019, is unique in collecting detailed latency data^21,22^. MyShake processes alerts in a parallelized manner, with users grouped into parallel alerting batches. For each unique incoming ShakeAlert update, each batch of users is processed by a single subprocess; multiple subprocesses run in parallel. For each batch of users, there is one subprocess run per alert update received. We term these subprocesses alerting runs. Each run is a thus an independent realization of the process by which latency is generated  (see Methods and Supplementary Text for more details). We develop a statistical characterization of alert delivery latency for use in our warning time simulations. We use a large dataset of MyShake alert latency that covers alerting runs across 33 events for which MyShake sent at least 10,000 alerts, spanning three orders of magnitude in number of alerts sent. We examine trends in latency with the number of alerts sent and the shape of the latency distribution.

Fig. 3a) shows the trend of median MyShake alert delivery latency for each individual MyShake run, against the number of alerts sent. We observe median delivery latency increases as number of users per run increases, though with large scatter. We quantify this linear relationship (in log-space) and the amount of noise, using Bayesian modeling (see Methods and Supplementary Text). As Fig. 3a) shows, our mean model was tightly constrained.

Next, we examine the shape of MyShake delivery latency distributions. Latencies result from a stochastic, multi-part process (MyShake alert processing, telecommunication delays, handoff to various message brokers). However, data from multiple events and runs (Figs. S7 and S8), reveals two key characteristics: positive (right) skew and a long tail, consistent with prior work^21,22^. Therefore, we aggregate data across events into one dataset, whose distribution we plot in Fig. 3b). We developed statistical, parametric shape models using Bayesian modeling, using four candidate distributions that could potentially capture these characteristics and ensure strictly positive latency (see Methods and Supplementary Text). A loglogistic distribution (Fig. 3b, gold solid line), emerged as our preferred model.

We combined these parametric, data-driven statistical models to produce full alert delivery latency ($t_{delivery}$) distributions for our end-to-end warning time simulations. The linear model sets the median latency based the number of targeted users in each EPIC update (set by the area enclosed by the alert contour), while our shape model defines the full latency distribution for subsets of users targeted by each update (see Methods). Our simulation replicates MyShake’s highly parallelized alert delivery protocol and couples it with a data-driven, statistical description of latency to achieve realism in alert delivery.

### End-to-end warning time simulations

We next show the results of our realistic, end-to-end warning time simulations for the two mainshocks of the Kahramanmaraş sequence, for three alerting thresholds: MMI 3, 4, and 5. We select a subset of EPIC updates (highlighted points in the top and middle rows of Fig. 2a; see Methods for selection criteria) for dissemination.

#### Warning times in the Pazarcık Event

**Figure 4:**
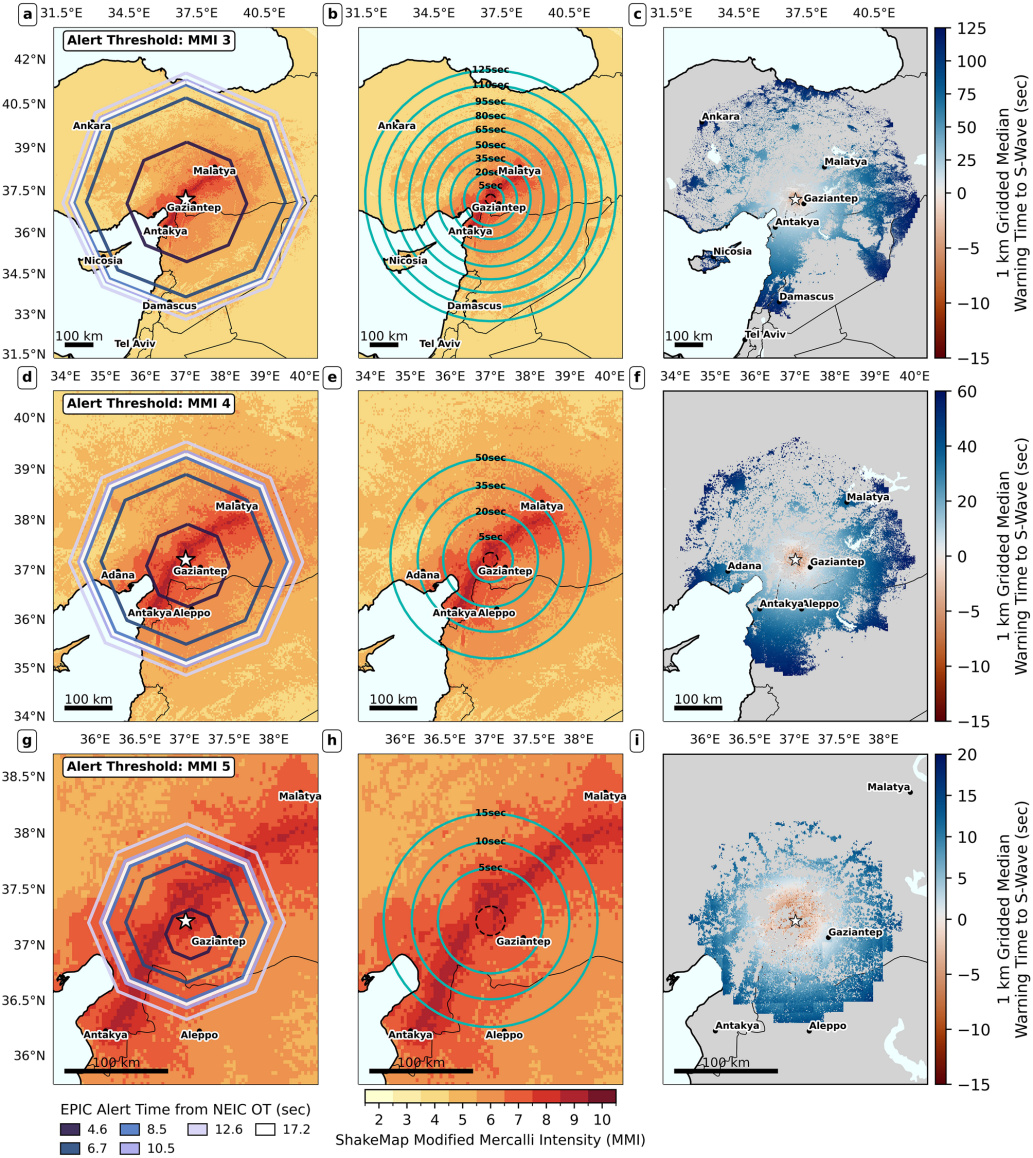
Temporal evolution of alerting contours and warning time-shaking distribution maps for the Pazarcık event across all three alerting thresholds. The first row, second, and third rows show maps for MMI 3, MMI 4; MMI 5, respectively. First column panels (**a**,**d**,**g**) show temporal evolution of alerting contours. Each contour is colored by the time the corresponding EPIC update was issued. Second column panels (**b**,**e**,**h**) show maps of median warning time contours. In each of these panels, warning time contours refer to the median warning time prior to the arrival of the S-wave. The contours are overlain on the USGS ShakeMap grid product for the event^25^. The late-alert zone (the area where the alerts are received after the estimated arrival of the S-wave), is enclosed by a dashed black circle, estimated from the 95th percentile of warning time. Alert time contours are estimated using interpolation of simulated alert receipts (see Supplementary Text and Fig. S8 for details). Third column panels (**c**,**f**,**i**) show median warning times, produced by aggregating raw user warning times onto a uniform grid of approximately 1 km resolution. Note the different geographic extents of the maps in the top, middle, and bottom rows. Note also that warning time contours are interpolated at 5 or 15 s intervals. As such, the alerted areas might extend past the maximum extent of these contours. The opposite is also possible, as our alerting scheme does not require Military Grid Reference System (MGRS) grid cells to be wholly contained within the alert polygon, but to just intersect it (see Figs. S10 and S11).

Figure 4 shows results for the Pazarcık event: Panels (a, c, e) show alert contour growth under the three simulated alerting thresholds. Panels (b, d, f) show median warning time contours to the initial S-wave, plotted atop the shaking distribution. The late-alert zone (the zone where alerts arrive after the S-wave, also called blind zone in the EEW literature; see dashed black circle) is very small (15–20 km), owing to EPIC’s rapid initial update (4.6 s from OT). The three scenarios share a common alerted area (compare Fig. 4a, c, e), with nearly identical warning times. For instance, Gaziantep (population of 2 million), which lies in the initial alert zone, receives about 5 s of warning under all three thresholds. In this near-epicentral zone, differences are driven purely by alert delivery latency; recall that our model implies longer delays when more users are targeted. Thus, MMI 5 alerting yields slightly longer warning times as fewer users are targeted compared to MMI 3 and 4.

MMI 5 alerting misses a large area, due to EPIC’s point-source assumption; without source finitenesss, the alert area does not grow large enough. Notably, Antakya receives no alerts under MMI 5 alerting, despite experiencing MMI 9+ shaking. Both MMI 3 and 4 alerting give Antakya around 35 s warning. Malatya, on the other end of the rupture in an area of MMI 8 shaking, gets $\sim$40 s warning (in initial MMI 3 alert area; second update for MMI 4 alerting). MMI 3 and 4 alerting have different maximum warning times and extent of alerted areas experiencing MMI 5 or weaker peak shaking. As MMI 3 alerting alerts a much larger area, the maximum warning time for MMI 3 alerting is $\sim$110 s, over double that under MMI 4 alerting ($\sim$50 s).

**Fig. 5:**
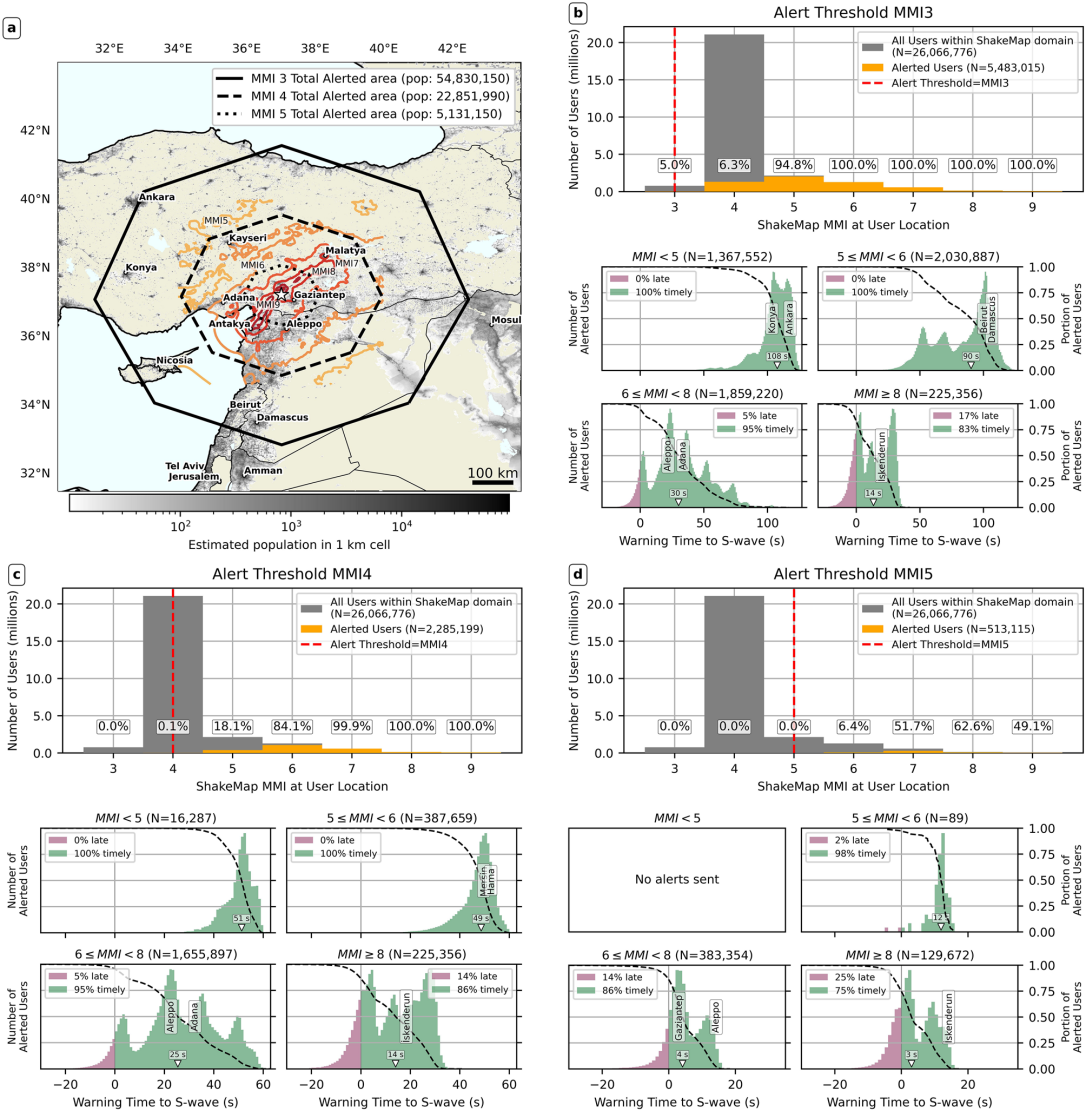
Total alerted areas, alert quality, and timeliness histograms for the Pazarcık event. (**a**) Population density, gridded onto a uniform geographic grid (30 arcsecond, or approximately 1 km resolution) and cumulative alerted areas for the three different alert thresholds: MMI 3, 4, and 5. We also plot simplified shaking intensity contours from USGS ShakeMap. (**b**) Alert quality and timeliness for MMI 3 alerting. (**c**) Alert quality and timeliness for MMI 4 alerting. (**d**) Alert quality and timeliness for MMI 5 alerting. Each of panels b, c, d, evaluates results for one of our three simulated alert thresholds (MMI 3, 4, and 5). In each panel, the top row shows the number of users experiencing a particular MMI level, binned in one MMI unit-wide bins, and how many users in each bin received an alert. Note that sampling of users at lower MMI levels might be incomplete (due to ShakeMap domain constraints). Simulated users are assigned an experienced MMI level by interpolating the USGS ShakeMap product^25,28^ for the two events using nearest-neighbor interpolation. The bottom row features four panels and assesses alert timeliness in four different MMI bins: $MMI < 5$ (moderate or weaker shaking), $5\le MMI < 6$ (moderate to strong shaking), $6\le MMI < 8$ (strong to very strong shaking), $MMI \ge 8$ (severe or stronger shaking). MMI bins are defined using the ShakeMap definitions of intensity, where an intensity unit encompasses observations at $\pm 0.5\,MMI$ units. Each panel plots the histogram (colored bars) as well as the empirical complementary cumulative density function (black dashed line) of S-wave warning times for users in each respective intensity bin. Note that these histograms are produced from the raw individual user warning times. The median warning time is marked with a white inverted triangle. For each intensity bin, we also plot the two most populous cities with population over 200,000 at their approximate warning time. Also note how the x-axis extent differs between the alert timeliness histograms for each alert threshold.

Figure 5 quantitatively analyzes these differences, showing the percentage of users alerted and alert timeliness across MMI intensity bins, using estimated intensities from the USGS ShakeMap to represent the peak shaking intensity experienced by simulated users. Under both MMI 3 and 4 alerting, practically all users experiencing $MMI\ge 7$ shaking are alerted (see top row, panels b-c), with similar timeliness (bottom row in panels b-c). However, under MMI 5 alerting, 52% of those experiencing MMI 7 shaking are not alerted (top row, panel d), and the percentage of timely alerts at $MMI\ge 8$ is halved (compare Fig. 5d, with b-c).

At the lower shaking intensities, the MMI 3 threshold ensures almost all users experiencing potentially damaging ($MMI\ge 5$) peak shaking were alerted, while MMI 4 alerting misses >80% of those experiencing MMI 5 peak shaking. All alerts sent under MMI 3 and 4 alerting to users experiencing $MMI\le 5$ were timely, but MMI 4 alerting misses large numbers of these users. Under the MMI 5 threshold, no users at $MMI\le 5$ are alerted due to EPIC’s magnitude underestimate.

#### Warning times in the Elbistan event

**Fig. 6:**
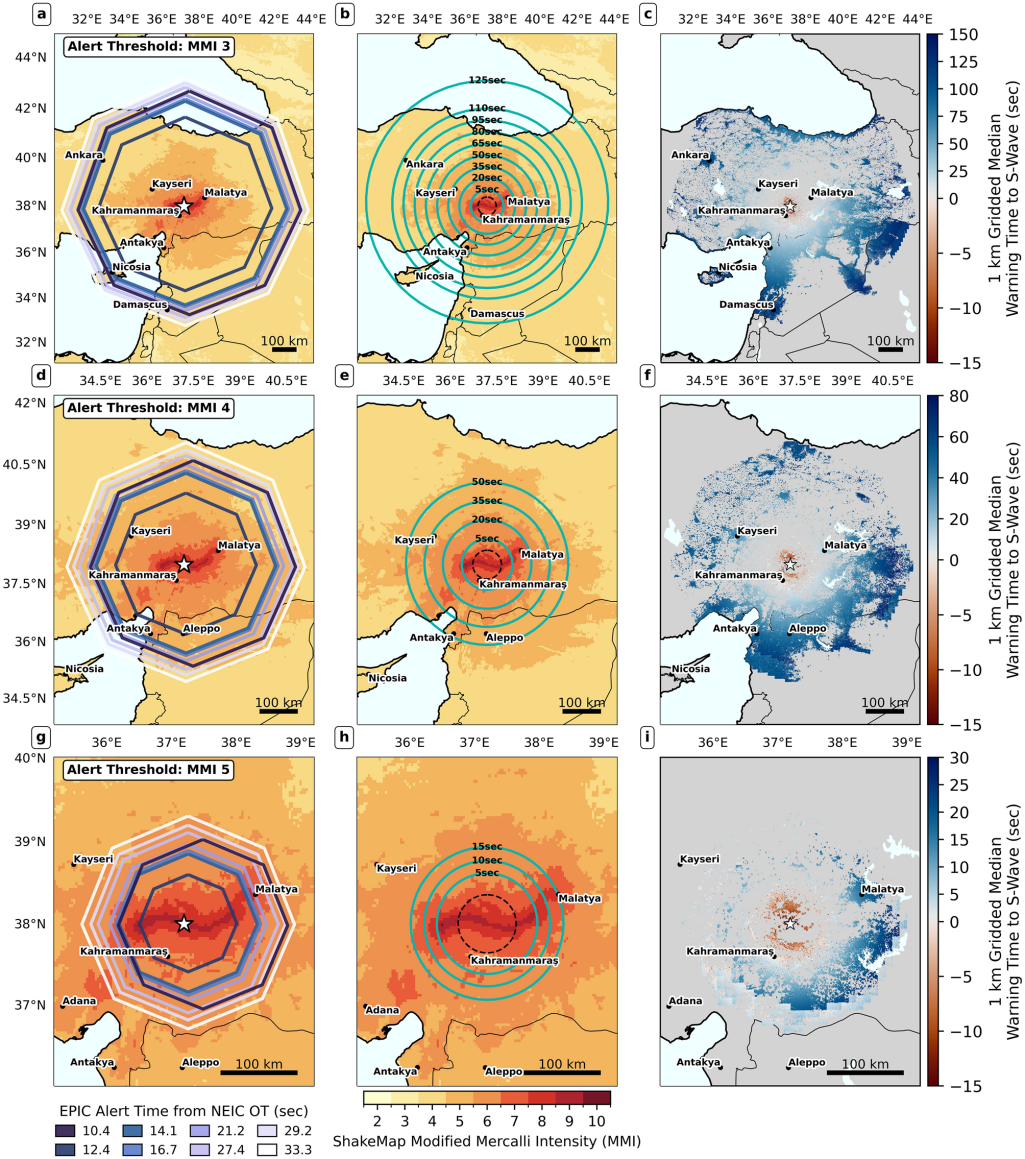
Same as Fig. 4, but for the Elbistan event. Note once again the different geographic extents of the maps in top, middle, and bottom rows. Note also that warning time contours are interpolated at 5 or 15 s intervals. As such, the alerted areas might extend past the maximum extent of these contours. The opposite is also possible, as our alerting scheme does not require MGRS cells to be wholly contained within the alert polygon, but to just intersect it.

**Fig. 7:**
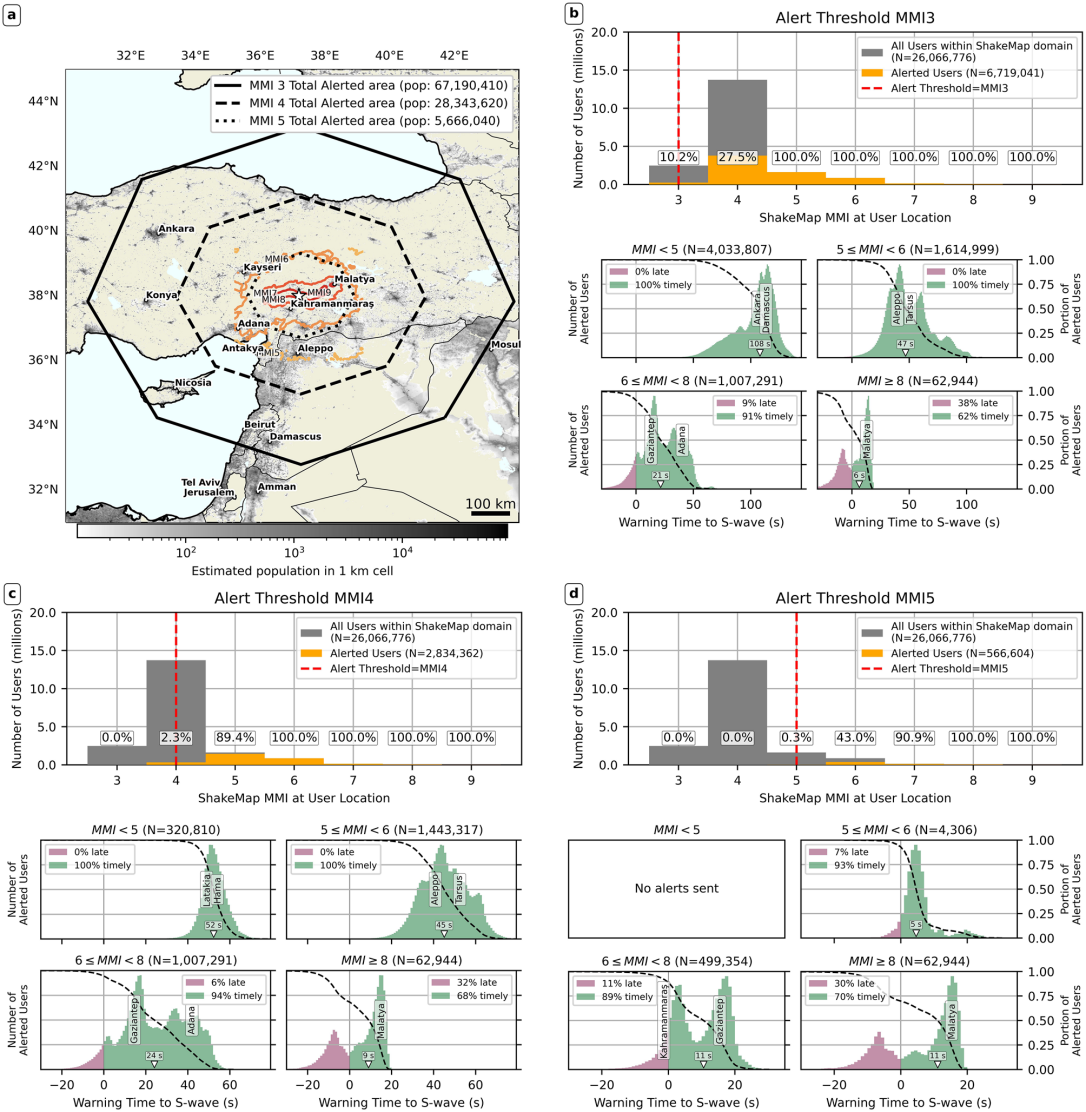
Total alerted areas, alert quality assessment, and timeliness histograms for the Elbistan event. The figure follows an identical structure to Fig. 5.

We show similar warning time and shaking distribution maps for the Elbistan event (Fig. 6). Three features contrast with the Pazarcık event (Fig. 4). First, alerted areas are much larger (+43% for MMI 3, +69% for MMI 4, +119% for MMI 5), and more people are alerted (+24%, +27%, and +10%, respectively) compared to the Pazarcık case. This is due to higher EPIC peak magnitude estimate (M6.72 vs M7.26). The MMI 3 cumulative alerted area (solid polygon in Fig. 7a) now extends well past Ankara. Second, alerting polygons for the first alert (darkest polygons in Fig. 6a, c, e) appear shifted towards the east with respect to the catalog epicenter, due to EPIC’s initial mislocation. This made warning times slightly longer towards the east (see spatial grids in Fig. 6c,f,i), as areas at the same epicentral distance got alerted earlier if they lay to the east of the true epicenter. Third, the size of the late-alert zone in panels b, d, f is much larger, as EPIC first alert took $\sim$10 s. While all users experiencing $MMI\ge 7$ shaking get alerted under all three thresholds (top rows, Fig. 7b-d), a large portion of these alerts are late; $\sim$30% of alerts arrived late with respect to the S-wave for those experiencing $MMI\ge 8$ peak shaking (bottom right plot Fig. 7b–d).

In terms of warning times (Fig. 6), Malatya, to the northeast of the rupture in an area of MMI 8 shaking, would receives $\sim$17 s of warning under MMI 3 and 4 alerting, being in the initial alert zone. It is only included in the initial alert area under MMI 5 alerting due to EPIC’s epicenter mislocation. To the north of Antakya, an isolated area of MMI 7 peak shaking (Çukurova plain) is included in the initial alert area under both MMI 3 and MMI 4 alerting (see Fig. 6 a and c), getting get 20-30 s of warning, but is mostly missed by MMI 5 alerting. The Turkish capital, Ankara, which experienced MMI 3-4 shaking, gets 90 s warning under MMI 3 alerting, but is missed under MMI 4 and 5 alerting.

Overall (Fig. 7), MMI 4 alerting warns all users experiencing $MMI\ge 6$ shaking, and only misses 11% of those experiencing MMI 5 shaking, similar to MMI 3 alerting. For both MMI 3 and 4 alerting, all alerts sent to users experiencing $MMI<6$ shaking arrive before the S-wave (Fig. 7b-d, second row). Timely alert percentages for users experiencing $MMI\ge 6$ shaking are also very similar. Median alert time for MMI 6-8 is $\sim 25\,s$ (inverted triangle in Fig. 7c and d, bottom left panel). Contrasts emerge for users experiencing MMI 4 peak shaking. MMI 3 alerting alerts 28% of those experiencing MMI 4 peak shaking, but MMI 4 alerting only alerts 2%. Under both thresholds, all alerts to users experiencing $MMI<5$ peak shaking are timely. Median alert times are larger for MMI 3 to MMI 4 alerting (107 s vs 70 s; compare left panel of the second row of Fig. 7b and c, as MMI 3 threshold alerts reach users further from the epicenter.

### Impacts of parameter choices: S-wave warning times and inclusion of latency

**Fig. 8:**
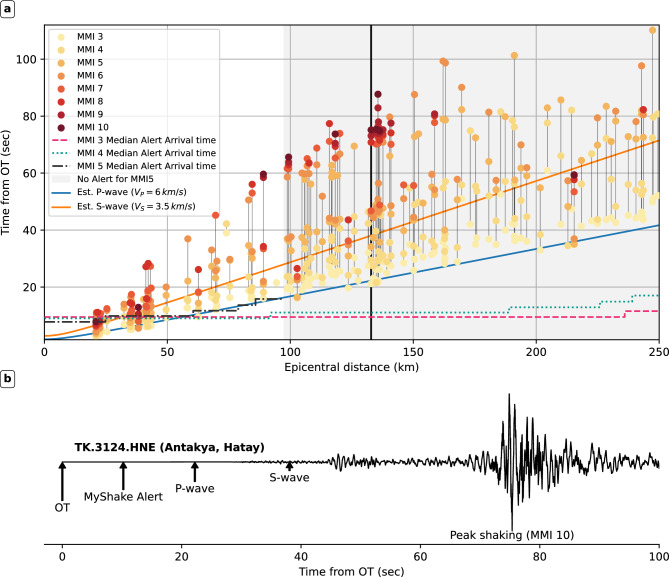
Chronology of alert arrivals and ground motions for the *M*7.8 Pazarcık earthquake. a) Chronology measured from origin time, plotted against epicentral distance. The time of first exceedance of MMI 3-9 for each of the TK network stations used in the replay, is shown as dots colored by MMI level. MMI is calculated from highpass filtered (above 50 s) acceleration timeseries and velocity timeseries using Equation (3) of Worden et al.^49^ (the arithmetic mean of converted MMI from velocity and acceleration). Horizontal lines indicate the alert arrival time at the station location, assuming median latency. It is estimated from the simulation data using the methodology outlined in the Supplementary Text and Fig. S10. Dashed line shows alert arrival times for MMI 3 alerting, dotted line shows arrival times for MMI 4 alerting, and dot-dashed line shows alert arrival for MMI 5 alerting. Note how at very small epicentral distances, alerts arrive almost at the same time under all three alerting thresholds, and how the MMI 3 and MMI 4 alerts are almost identical. The blue and orange lines indicate the P- and S-wave moveout using constant moveout velocities of 6 and 3.5 km/s, respectively. b) Acceleration waveform from the east component of station TK.3124 in Antakya, Hatay Province. The station is at an epicentral distance of 133 km, and its location is marked in panel a) by a solid black line. Timestamps for the event origin time, estimated P- and S-wave arrivals, the alert arrival, as well as time of peak shaking (PGA), are marked on the waveform.

In our simulation, we calculated S-wave warning times, as we did not have seismograms at every user location. However, where timeseries data is available, warning times relative to the time of exceeding particular MMI levels can be calculated. Figure 8a,b shows MMI exceedance times measured from Pazarcik earthquake waveforms. We see the S-wave tracking MMI4-5 shaking for epicentral distances beyond 50 km. Warning times to MMI 6 and peak shaking are generally longer than inferred from S-wave arrivals. The Elbistan event also shows this (Figure S14), although these time intervals are shorter. In the Antakya waveform example (TK.3124, Fig. 8b), S-wave warning time is 25 s for MMI 3 and 4 alerting, but the time until peak shaking is 64 s. We also observe MMI 5 alerting completely misses sites experiencing MMI 8 peak shaking (see also Figures S15 and S16). In large earthquakes, some users may receive sufficient warning to take protective action before the most intense shaking, even if they have felt the initial shaking before alert arrival. This is valued by EEWS users as a means of mental preparation and confirmation of an earthquake already under way^1,50^. Figure 8 shows the first exceedance of MMI 3 (generally felt) shaking tracks the first-arriving P-wave, especially beyond 80 km, where alerts can outpace the P-wave. If the P-wave is felt, it is very likely the S-wave will bring significant (MMI 6+) shaking^50^. For users getting an alert then feeling the P-wave, the alert can again confirm an evolving earthquake.

We also quantify the impact of incorporating alert delivery latency on warning times, by repeating the analysis of Fig. 5 and 7, but omitting latency altogether (Fig. S13). Pronounced contrasts emerged in the warning time distributions for both events. For instance, in the Pazarcık event, all sent alerts, across all three thresholds, were timely. However, key trends of increasing median warning times for users experiencing weaker shaking persist. The locations of the peaks in the histograms in Fig. S13 are purely controlled by where high population density areas are relative to the epicenter; both an alert and the S-wave hits everyone in a particular region at roughly the same time. These peaks persist in Figs. 5 and 7, but are broadened by alert delivery latencies. As expected, incorporating latency not only shortens warning times, but also vastly increases their variability.

## Discussion

Our simulations illuminate important aspects of EEWS end-to-end performance in large onshore crustal earthquakes near large populations. Firstly, they underscore the importance of commonly used point-source algorithms like EPIC. With a dense seismic network, EPIC can alert extremely quickly (e.g., 4 s for the $M_W7.8$ Pazarcık earthquake), with early magnitude estimates tracking initial rupture growth. Our EPIC replays demonstrate that EEWS analyses assuming perfect source characterization, while informative^16,18^, do not reflect real-time complexities, like initial mislocation (e.g., Elbistan event). The edge-of-network Yayladagi event underscored EPIC’s struggles with locating such events accurately. Williamson et al.^36^ showed pre-conditioning EPIC’s location scheme with prior seismicity in edge- or out-of-network areas improves location accuracy, in turn improving magnitude estimates.

Our results confirm EPIC’s tendency to underestimate the magnitude of large events, due to only using 4 seconds of P-wave data. Extending the window to the estimated S-wave arrival, another point-source approach^38^ estimated Pazarcık at *M*7.5 in real-time. Nonetheless, EPIC’s early estimates were $M\,\sim 7$ for both Kahramanmaraş mainshocks, despite their different source characteristics. With MMI 3 or 4 alerting thresholds, alerts in our simulations quickly reached large areas, providing up to 20-30 s warning in areas of MMI6-8 peak shaking. This would provide ample time for individuals to Drop, Cover, Hold On (this takes a median of 9 s^19,51^). By contrast, MMI 5 alerting performed significantly worse. It missed many users experiencing MMI 6 and 7 shaking, due to EPIC’s magnitude saturation and point-source assumption. These conclusions align with past EPIC-only analyses^52^ and analyses considering all algorithms in ShakeAlert^19^. Given California’s comparable potential for Kahramanmaraş-type events, our results suggest a minimum MMI 4 alerting threshold for ShakeAlert-MyShake alerts would achieve identical performance to the current MMI 3 threshold for those experiencing damaging shaking in large earthquakes.

EPIC’s rapid initial reporting led to warning times in our simulation that compare favorably with results from the finite source FinDer algorithm^37^, which complements EPIC in ShakeAlert. FinDer surpassed EPIC’s magnitude estimates after $\sim$10 s, but it benefits from defining the source as a finite line rather than a point, extending alert areas. While direct comparison with Böse et al.^37^ is limited by different configurations (ground-motion models, definitions of warning times), we show similar per-station warning times to MMI 6 arrival in Fig. S15 (Pazarcık) and S16 (Elbistan). Considering stations within 50 km of the Pazarcık epicenter, we show 5-10 s warning, compared with up to 5 s warning in FinDer replays. These would drop to near zero accounting for 5 s delivery latency. Results for Antakya are similar under MMI 3-4 alerting; under MMI 5 alerting, EPIC does not alert Antakya, while FinDer provided 20 s of warning before MMI 6 shaking. QuakeUp point-source replays^38^ reached similar conclusions; their lower MMI 4 alerting threshold achieved longer warning times ($\sim 20\,s$ at $\sim 100\,km$ distance) and a more favorable ratio of successful-to-missed alerts compared to their higher MMI 6 threshold.

At large distances, our EPIC-only simulations show only the MMI 3 threshold alerted substantial numbers of users who experienced light or weaker ($MMI<5$) shaking, though 50% were still missed. Alerted users experiencing $MMI<5$ shaking had very long S-wave warning times under MMI 3-4 alerting (>1 minute). EPIC’s point-source alert areas cannot grow sufficiently to reach the majority of users experiencing weaker ($MMI<5$) shaking. Algorithms that do not suffer from magnitude saturation (e.g., FinDer, geodetic algorithms like GFAST-PGD^53^), can better reach these users. Whether these users should be alerted remains an open question for alert providers. Public dissatisfaction at missed alerts for MMI 4 shaking in the Los Angeles Basin during the 2019 Ridgecrest M7.1 earthquake prompted a lowering of alert thresholds to extend alerting areas further^35^. At the same time, lowering thresholds can result in frequent alerts for small earthquakes, targeting users who do not feel shaking, potentially resulting in “alert fatigue”^54^. Optimizing EEWS performance in infrequent large vs frequent small events is a delicate balance, and warrants further work.

Our simulation results also highlight the importance of considering alert delivery latencies when assessing EEW performance. While not an issue for machine-to-machine delivery, latencies are critical for public alerting. While the MyShake alert delivery protocol is by no means the only way to deliver alerts, reproducing it makes alert delivery in our simulation realistic. Latencies not only reduce warning times, but also introduce significant variability – a key feature of any realistic, latency-aware simulation. Our data-driven statistical model provides one avenue for incorporating this variability. Even after accounting for latency, our results show that a network-based EEWS could have provided actionable ($>10\,s$) warning times for many who experienced damaging shaking during the Kahramanmaraş earthquakes.

Although our results represent hypothetical performance in Türkiye for these two specific events, our framework is flexible and extensible. It could simulate performance across various scenarios using real-world or simulated alert progressions, using individual algorithms or algorithm combinations, or alternative ground-motion forecast logic^55^. It could also be run over whole catalogs to produce probabilistic warning time maps^56^. Most importantly, our simulation yields realistic and illustrative results that can help calibrate EEWS expectations among stakeholders, especially for EEWS using point-source algorithms like EPIC.

## Methods

### EEW Replay Methodology

#### EPIC Point Source Algorithm

We replayed the processed waveform data in simulated real time through the production version (i.e. making no changes) of the point source network early warning algorithm Earthquake Point-source Integrated Code (EPIC) currently used in the US ShakeAlert EEWS^10,12^, to obtain real-time estimates of epicentral location and magnitude. The EPIC algorithm consists of four parts: triggering, association, location, and magnitude determination.

EPIC triggers on P-wave arrivals in seismic waveform data using a short-term/long-term average (STA/LTA) triggering logic^57^. It then associates available triggers into events. Associated triggers are used to compute an epicentral location and origin time using a grid search in geographic space, assuming a fixed depth of 8 km.

The computed location is used to estimate magnitude using peak displacement amplitudes ($P_d$) from vertical component seismograms, using up to 4 seconds of data. $P_d$ amplitudes are converted into individual station magnitudes ($M_{Pd}$) using the global scaling law of Kuyuk and Allen^58^. Individual station magnitude estimates are then averaged, weighted by the amount of available data at each station^35^, to determine a single EPIC magnitude estimate for the event. EPIC will issue an alert as soon as at least four unique triggers are associated into a single event and all quality criteria are passed. As more data becomes available, EPIC assimilates it in real-time and revises its real-time source estimates at a maximum frequency of one update every second. Further details on EPIC are provided in the Supplementary Text.

#### Waveform data

For the replay through the EPIC point source algorithm, we used available broadband and strong motion data from the Kandili Observatory Regional Network, KO^59^ and the Turkish National Strong Motion Network, TK^24^. Our dataset was almost identical to that used by Böse et al.^37^ in their replays of the Kahramanmaraş sequence using the FinDer finite fault algorithm. Böse et al.^37^ reported that a substantial number of stations had significant data gaps or instrumental glitches. Despite of this, we did not discard any data to better align with a real-time operational situation. We also chose to use broadband records (HH channels), even though all available broadband records were clipped, given that the production version of EPIC in the US ShakeAlert system ingests both strong motion and broadband data.

### MyShake latency modeling

In order to perform an end-to-end simulation of warning times in the mass public alerting case, we incorporated alert delivery latencies by using a statistical model. We develop our model using real-world alert delivery latency data collected by the MyShake platform. MyShake has been publicly delivering alerts from the USGS ShakeAlert system since October 2019, delivering over 4 million alerts for over 140 events on the US West Coast^22^.

MyShake processes updates from the ShakeAlert system in parallel, with users randomly partitioned into batches to speed up processing. Not all users in a batch are necessarily alerted during a given alerting run, as only users within the alert area receive the alert. We define an alerting run as one such subprocess delivering a single alert update to the subset of users in its assigned batch whose locations lie within the alert area. Each alerting run therefore represents an independent delivery event with its own latency characteristics. Latency statistics are available per user and also aggregated on a per-run basis^21,22^.

Previous analyses of MyShake latency data have shown that the time taken for MyShake to process a ShakeAlert update varies depending on the total number of users targeted in each run^21,22^. We thus sought to statistically reproduce the characteristics of delivery latencies, namely the trend of run median latency with number of alerts sent and the shape of the latency distribution.

We first ran a Bayesian linear regression to model the relationship between the median delivery latency for a given MyShake run and the total number of users targeted by that run. In our Bayesian model, we also obtained an estimate of the statistical variability about our linear model (see Supplementary Text).

We then modeled the shape of the latency distribution. We observed that across many events, latency distributions (see Figs. S7 and S8) were generally similar, showing right-skewed shapes with a very wide tail. We therefore combined all alert delivery latency data into a single non-dimensional dataset for modeling the shape of the latency distribution using parametric distribution models. Latency observations were non-dimensionalized by dividing each observation the median latency of its MyShake alerting run.

We sought a distribution that captured the right-skewness and long tails, as well as ensuring positivity (as latency cannot be negative; this would imply that a user can get an alert before it is issued by the EEW system). We tested four candidate distributions within a Bayesian framework: a Weibull model, a Gamma model, a lognormal model, and a loglogistic (Fisk) model. We quantified the fit using the Watanabe-Akaike Information Criterion and the Kullback-Leibler divergence as measures of fit, as well as posterior predictive check plots as visual checks. Based on these, we selected the loglogistic model as our preferred latency shape model. We provide further details in the Supplementary Text.

### Warning Time Simulation

Using the results from our EPIC replays for the Pazarcık and Elbistan events, and our alert delivery latency statistical model, we set up our realistic end-to-end warning time simulation. The simulation features multiple steps to assemble the components of warning time, $t_{warn}$, outlined in Equation 1: time of alert issuance ($t_{alert}$), alert delivery latency ($t_{delivery}$), and the time of arrival of strong ground shaking, approximated by the arrival of the first S-wave ($t_S$).

Our simulation first requires a set of users for which to simulate warning times. We account for real-world targeted user counts, assuming 10% of the population in the area had access to a MyShake-like smartphone alert delivery platform. This is in line with current levels of user penetration in California ($\sim 6.5\%$^22^). We sample user locations in our domain of interest using a high-resolution population counts dataset^39^.

In our simulation workflow, $t_{alert}$ is set by the time-evolutive EPIC source estimate. Selected updates from EPIC are passed to a MyShake alert delivery latency simulator, with the update time marked as $t_{alert}$. Updates are selected such that each one represents a significant change in source parameters over the previous one, mimicking an alert Decision Module like the one in ShakeAlert^10,19^. This is the way that MyShake receives updates from ShakeAlert in real-world operation.

We convert EPIC’s simulated real-time magnitude estimate into an alerting extent for our three alerting thresholds (MMI 3, 4, 5). We define the extent of alert contours using the production ShakeAlert magnitude lookup tables, mimicking the behavior of the eqinfo2GM module^45^. In these tables, the extent of shaking at each MMI level from 2 to 9 is defined for earthquake magnitude M3-M8.5. It is estimated as the average of the extent obtained from peak ground acceleration (PGA) and velocity (PGV) using a combination of the ground-motion-to-intensity conversion equation (GMICE) of Worden et al.^49^ and the median PGA and PGV predictions from the Boore and Atkinson (BA08) ^60^ ground-motion model (GMM). We deem the BA08 GMM adequate for this active crustal region, as it is an active crustal GMM derived in part using data from Türkiye. Alert extents defined using the NGA-W2 suite^61^ of GMMs are not significantly different. The alert extent tables are compiled for a source depth of 8 km and a $V_{S30}$ (time-averaged shear wave velocity in the top 30 m) of 500 m/s^45^. We convert the alert extent in kilometers to octagonal alerting polygons, following ShakeAlert practice^45^. These polygons are then used to alert users within the simulation domain. The time at which an alert contour intersects the 10x10 km MGRS cell containing a user’s location is marked as $t_{alert}$ for that user (see Equation 1).

To simulate alert delivery latencies ($t_{delivery}$ in Equation 1), we generate a distinct latency distribution for each alerting run, defined previously as the delivery of a single alert update to users in a batch whose MGRS locations intersect the alert contour. The simulation then proceeds in two steps for each run.

First, we simulate the run’s median latency. This is calculated by evaluating our linear model for the number of users in the batch. We add noise to this median estimate by adding a random variate drawn from the model’s posterior noise distribution. Second, we generate the full latency distribution for all users in the run. To do this, we draw random samples from our log-logistic shape model–using parameters sampled from their posteriors–and rescale the resulting non-dimensional latency samples back to physical units by multiplying them by the simulated median latency.

In line with the real-world operation of MyShake, simulated users are pre-assigned into batches of approximately 100,000, and alert updates for each batch are processed in parallel by a single run. This design ensures our simulation mirrors the system configuration that produced the data used to train the statistical model. While we simulate alerting about 10% of the population, in line with current levels of user penetration in California^22^, we expect results to generalize to larger numbers of users provided the level of parallelism and load per batch (i.e., no more than 100,000 users per batch) is maintained at current levels.

The final, event-wide latency distribution is therefore the aggregate mixture of the individual distributions generated across all batches and all alert updates (all alerting runs). Alert targeting is done in parallel for each batch, by determining the 10x10 km MGRS cells containing users that intersect the alert contour.

Finally, we compute warning times for each user considered in the simulation by comparing the total time taken for the alert to be delivered (as shown in Equation 1) to the estimated time of S-wave arrival at the user’s location, assuming a constant S-wave moveout velocity of 3.5 km/s. These warning times therefore incorporate all possible sources of delay (e.g., data packetization and packet queueing, picking delays, calculation time etc., see^62^) apart from data transfer times, which we assume negligible. For state-of-the-art networks, like the one in California, data transfer times are $\le 1\,s$ under normal operations^63^, and while they can can increase under strong shaking, remain low for P-wave packets^32^.

For a more detailed discussion of the simulation methodology, see the Supplementary Text.

## Supplementary Information

Supplementary Information.

## Data Availability

Waveform data from the TK and KO networks can be downloaded at https://tdvms.afad.gov.tr/continuous_data. We used population count data at 1 km resolution from the 2020 release of the WorldPop database^39,64^, available at https://www.worldpop.org/datacatalog/. The MyShake latency dataset used is available as a Zenodo repository^22,65^. We obtained event data products by querying the USGS Comprehensive Earthquake Catalog (ComCat), available at https://earthquake.usgs.gov/data/comcat/ (last accessed January 2025), using the Python package libcomcat^66^. Maps use free vector and raster map data available at naturalearthdata.com, as well as topographic data from the General Bathymetric Chart of the Oceans^67^.

## Code availability

The EPIC software is not publicly released. Bayesian statistical modeling and analysis was performed in Python using the PyMC^68^ and arviz^69^ packages. Figures were compiled using the matplotlib^70^ and CartoPy^71^ packages in Python. Our end-to-end warning time simulation algorithm is available as a Python code, along with example inputs and outputs, via a . Zenodo repository.
