# Supplementary material for: Exploring end-to-end earthquake early warning performance in large earthquakes using the February 2023 Kahramanmaraş, Türkiye sequence
Source: Sci Rep. 2025 Dec 12;16:327. doi: 10.1038/s41598-025-29755-z (PMC12770601; doi:10.1038/s41598-025-29755-z)
Supplement: Supplementary file 1 — Supplementary Information. [file 41598_2025_29755_MOESM1_ESM.pdf]

Supplementary Material for

Exploring End-to-End Earthquake Early Warning Performance in Large Earthquakes Using the February 2023 Kahramanmaraş, Türkiye Sequence

Savvas Marcou, Angela I. Lux, Andrei Akimov, Amy L. Williamson, and Richard M. Allen

Contents of this file

- Supplementary Text
- Figures S1 to S15
- Table S1 to S2

EPIC Replays

EPIC Initial Location and Magnitude Estimates

EPIC is a point source algorithm that aims to estimate an epicentral location and magnitude for an earthquake underway using real-time P-wave data from seismic stations. It will issue alerts (i.e., push its source estimates to a solution aggregator or decision module[1,2] that decides whether to issue alerts to users and which alerts should be sent), only when four or more stations have been associated into an event, and when an "update" (a single EPIC source estimate) passes a series of quality control criteria (magnitude and location within acceptable ranges, percent of active stations triggering etc.). More details are given in previous publications about EPIC and ShakeAlert[3–7].

EPIC determines a location, and subsequently a magnitude estimate in real-time by incorporating P-wave triggers contributed by an STA/LTA triggering algorithm. Triggers are checked against a variety of criteria such as a horizontal-to-vertical ratio check and various teleseismic filters[4]. Checked triggers are associated into events subject to proximity and travel-time window criteria, and used to determine an event location. When only one trigger is available, the location is set to the location of the triggered station. With two triggers from two different stations, the preliminary location moves to a point between the first and second station. Once provided with triggers from at least four unique stations, EPIC uses a direct grid search to determine the best-fitting event location[4–6]. This grid search uses a fixed depth of 8 km and a 1D velocity model. EPIC will calculate a location using only the first 10 triggers associated with a particular detection, to minimize processing times.

Once a location is available, EPIC will attempt to calculate a magnitude using highpass-filtered (0.075 Hz) vertical component peak displacement (P_d) amplitudes using as much as 4 seconds of data after the trigger. P_d amplitudes are converted into individual station magnitudes (M_{Pd}) using the global $M_{Pd} - P_d$ scaling law of Kuyuk and Allen (KA13)[8]. The overall EPIC real-time magnitude estimate is calculated using a weighted average of all available individual station magnitudes, weighted by the amount of data available at each station (with the maximum capped at 4 seconds of data). For data from a particular station to be used, the available P_d amplitude must lie within an acceptable P_d window, have a signal-to-noise ratio (SNR) of 0.5 or greater (defined by comparing peak amplitudes within the P_d window with those from 30 s before the trigger), and the station must be within 200 km of the estimated epicenter. This means that if certain stations pass the trigger criteria (meaning they can be associated into events and contribute to location estimates), but fail the P_d checks, an alert issued by EPIC might have a magnitude may be calculated using less than four individual station magnitudes. Furthemore, as the KA13 scaling law includes a term for epicentral distance, a large location error might have an adverse impact on the estimated magnitude[6].

To aid the reader in the interpretation of initial EPIC location and magnitude estimates, we provide snapshots of EPIC's source estimates and the location of contributing stations for the three events in Figures S1 (Pazarcık event), S2 (Elbistan event), and S3 (Yayladagi event), as well as full tables of all EPIC alerts issued for these three events (Tables S1, S2, S3).

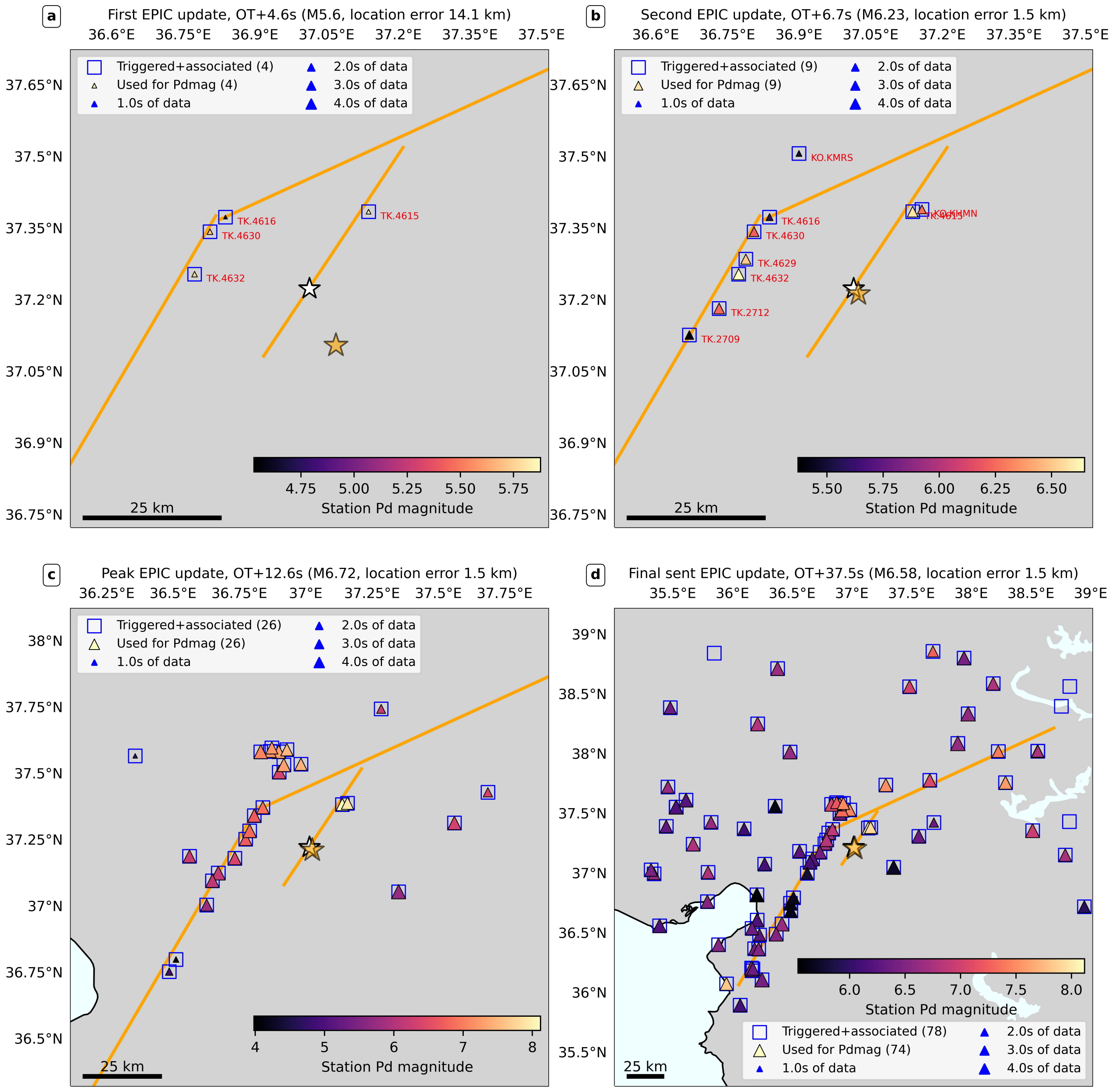

Figure S1: Trigger maps for individual EPIC updates in the Pazarcık event replay. a) Initial EPIC update. b) Second EPIC update. c) Peak magnitude EPIC update. d) Final EPIC update selected for dissemination. In each panel, the title indicates the magnitude estimate, location error and update time after the NEIC origin time. Each panel shows all stations triggered and associated into the EPIC event as blue squares. Stations that contributed to the magnitude estimate have a colored triangle plotted within the square. The triangle is colored by the station individual M_{Pd} value, and sized by the amount of data available at the time of the update. The EPIC epicenter estimate is shown as a gold star. We also plot the NEIC catalog epicenter as a white star. Note that the geographic extent changes between the four panels.

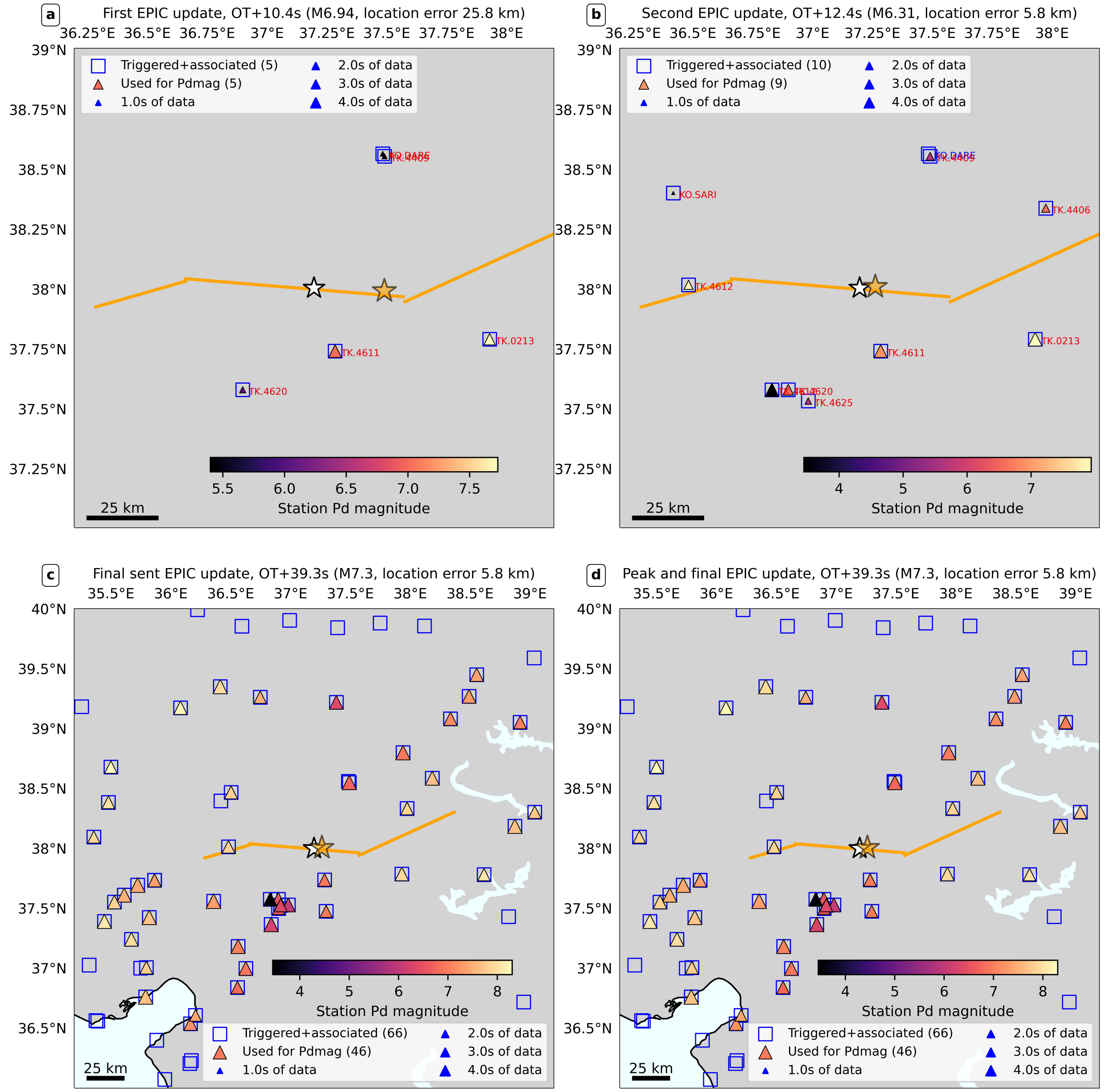

Figure S2: Trigger maps for individual EPIC updates in the Elbistan event replay. a) Initial EPIC update. b) Second EPIC update. c) Final EPIC update selected for dissemination. d) Peak magnitude and final EPIC update. Panels follow the same structure as Figure S1.

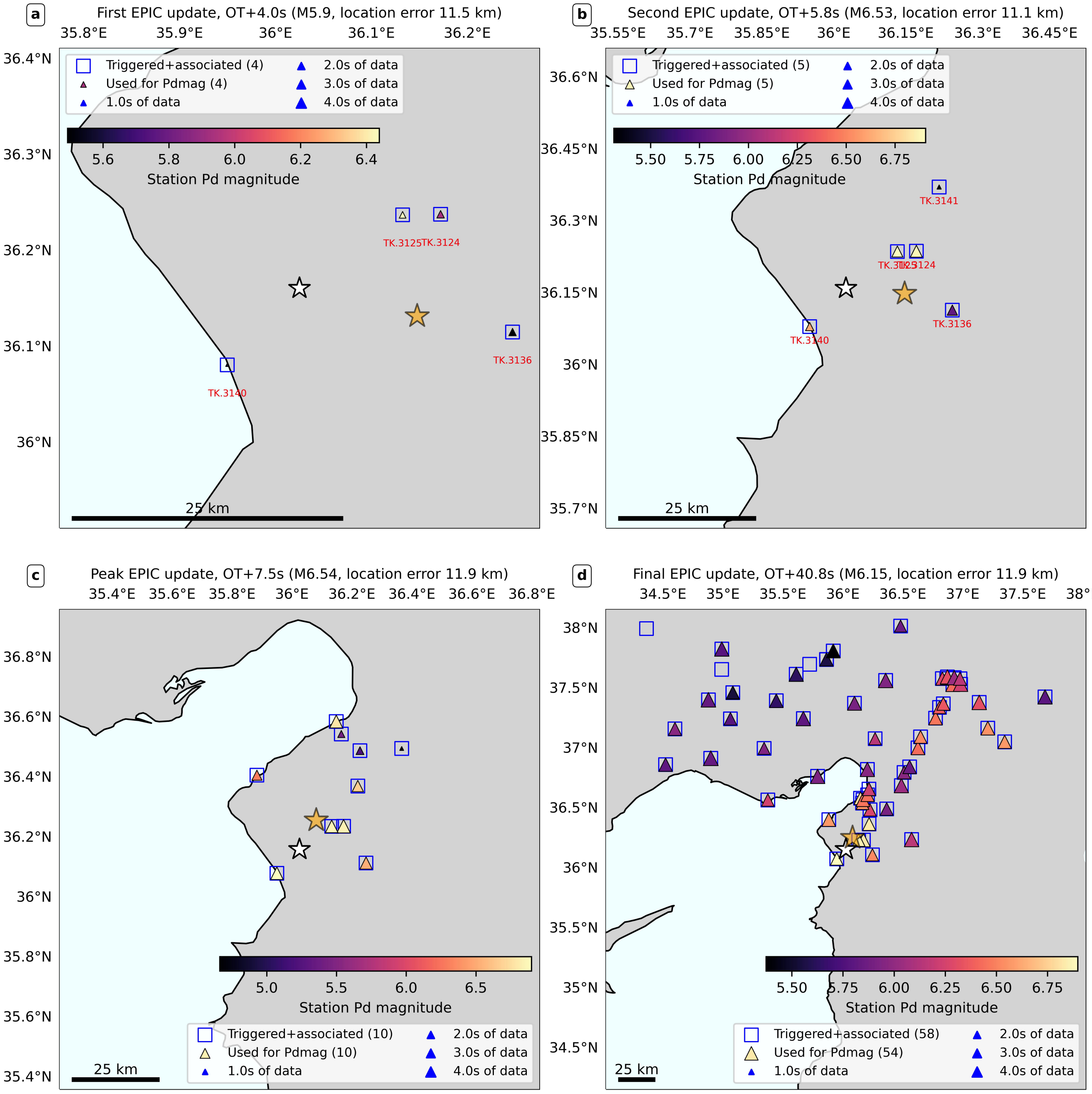

Figure S3: Trigger maps for individual EPIC updates in the Yayladagi event replay. a) Initial EPIC update. b) Second EPIC update. c) Peak magnitude EPIC update. d) Final EPIC update. Panels follow the same structure as Figure S1.

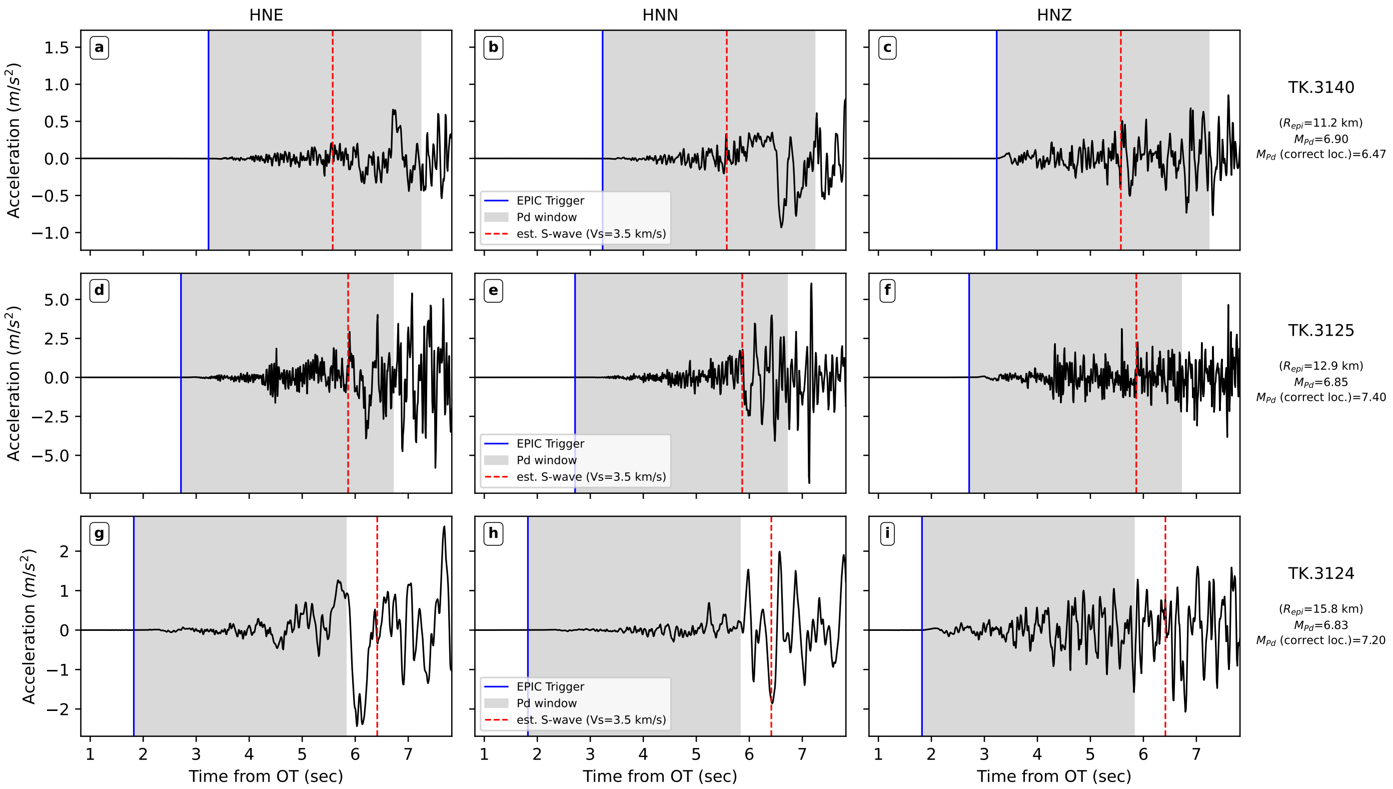

Figure S4: Three-component waveform plots for the three closest stations used by EPIC in the Yayladagi event (stations TK.3140, TK3125, TK3124). Each row shows one station, labeled with its EPIC P_d magnitude estimate (M_{P_d}) its M_{P_d} if the location were estimated correctly (correct loc.), and the distance between the station and the NEIC estimated epicenter (R_{epi}). Note that these stations were all within 16 km of the epicenter. Each column plots one component: the left column plots the east component, the middle column plots the north component, and the right column plots the vertical component (used for triggering and amplitude measurements). The timing of the EPIC P-wave trigger is marked on all waveforms by a vertical blue line, while the estimated first S-wave arrival based on the NEIC origin time and assuming a moveout velocity of 3.5 km/s is shown as a red dashed line. S-wave energy can be seen in the P_d window for all three stations. It manifests as a marked shift towards lower frequencies in the wavetrain and an increase in amplitudes. This is particularly pronounced for the east component (HNE), but it is also seen in the vertical (HNZ), which is used for extracting P_d measurements for EPIC's magnitude estimation.

Alert Time (UTC)	EPIC Latitude	EPIC Longitude	EPIC depth (km)	EPIC Magnitude	EPIC Origin Time (UTC)	Location Error (km)	Alert Time after Event Origin (seconds)	Alert Time after First Update (seconds)	MMI3 alert radius (km)	MMI4 Alert Radius (km)	MMI5 Alert Radius (km)
2023-02-06 01:17:39	37.1068	37.0695	8	5.60	2023-02-06 01:17:32	14.1	4.6	0.0	236	92	25
2023-02-06 01:17:41	37.2147	37.0243	8	6.23	2023-02-06 01:17:33	1.5	6.7	2.1	392	189	61
2023-02-06 01:17:43	37.2147	37.0243	8	6.49	2023-02-06 01:17:33	1.5	8.5	3.9	441	226	79
2023-02-06 01:17:45	37.2147	37.0243	8	6.58	2023-02-06 01:17:33	1.5	10.5	5.9	458	239	86
2023-02-06 01:17:47	37.2147	37.0243	8	6.72	2023-02-06 01:17:33	1.5	12.6	8.0	484	260	97
2023-02-06 01:17:50	37.2147	37.0243	8	6.69	2023-02-06 01:17:33	1.5	15.2	10.6	479	256	95
2023-02-06 01:17:52	37.2147	37.0243	8	6.54	2023-02-06 01:17:33	1.5	17.2	12.6	450	233	82
2023-02-06 01:17:53	37.2147	37.0243	8	6.55	2023-02-06 01:17:33	1.5	18.9	14.3	452	234	83
2023-02-06 01:17:55	37.2147	37.0243	8	6.53	2023-02-06 01:17:33	1.5	21.2	16.6	448	231	82
2023-02-06 01:17:57	37.2147	37.0243	8	6.55	2023-02-06 01:17:33	1.5	23.1	18.5	452	234	83
2023-02-06 01:18:00	37.2147	37.0243	8	6.52	2023-02-06 01:17:33	1.5	25.2	20.6	447	230	81
2023-02-06 01:18:02	37.2147	37.0243	8	6.54	2023-02-06 01:17:33	1.5	27.2	22.6	450	233	82
2023-02-06 01:18:04	37.2147	37.0243	8	6.56	2023-02-06 01:17:33	1.5	29.3	24.7	454	236	84
2023-02-06 01:18:06	37.2147	37.0243	8	6.55	2023-02-06 01:17:33	1.5	31.4	26.8	452	234	83
2023-02-06 01:18:08	37.2147	37.0243	8	6.55	2023-02-06 01:17:33	1.5	33.5	28.9	452	234	83
2023-02-06 01:18:10	37.2147	37.0243	8	6.57	2023-02-06 01:17:33	1.5	35.3	30.7	456	237	85
2023-02-06 01:18:12	37.2147	37.0243	8	6.58	2023-02-06 01:17:33	1.5	37.5	32.9	458	239	86
2023-02-06 01:18:14	37.2147	37.0243	8	6.58	2023-02-06 01:17:33	1.5	39.5	34.9	458	239	86
2023-02-06 01:18:16	37.2147	37.0243	8	6.58	2023-02-06 01:17:33	1.5	42.0	37.4	458	239	86
2023-02-06 01:18:18	37.2147	37.0243	8	6.58	2023-02-06 01:17:33	1.5	44.1	39.5	458	239	86
2023-02-06 01:18:21	37.2147	37.0243	8	6.58	2023-02-06 01:17:33	1.5	46.2	41.6	458	239	86
2023-02-06 01:18:22	37.2147	37.0243	8	6.58	2023-02-06 01:17:33	1.5	48.1	43.5	458	239	86
2023-02-06 01:18:25	37.2147	37.0243	8	6.58	2023-02-06 01:17:33	1.5	50.2	45.6	458	239	86
2023-02-06 01:18:27	37.2147	37.0243	8	6.58	2023-02-06 01:17:33	1.5	52.8	48.2	458	239	86
2023-02-06 01:18:29	37.2147	37.0243	8	6.58	2023-02-06 01:17:33	1.5	54.7	50.1	458	239	86
2023-02-06 01:18:31	37.2147	37.0243	8	6.58	2023-02-06 01:17:33	1.5	56.6	52.0	458	239	86
2023-02-06 01:18:33	37.2147	37.0243	8	6.58	2023-02-06 01:17:33	1.5	58.7	54.1	458	239	86
2023-02-06 01:18:35	37.2147	37.0243	8	6.58	2023-02-06 01:17:33	1.5	60.7	56.1	458	239	86
2023-02-06 01:18:37	37.2147	37.0243	8	6.58	2023-02-06 01:17:33	1.5	62.7	58.1	458	239	86
2023-02-06 01:18:40	37.2147	37.0243	8	6.58	2023-02-06 01:17:33	1.5	65.4	60.8	458	239	86
2023-02-06 01:18:42	37.2147	37.0243	8	6.58	2023-02-06 01:17:33	1.5	67.3	62.7	458	239	86
2023-02-06 01:18:43	37.2147	37.0243	8	6.58	2023-02-06 01:17:33	1.5	69.2	64.6	458	239	86
2023-02-06 01:18:45	37.2147	37.0243	8	6.58	2023-02-06 01:17:33	1.5	71.2	66.6	458	239	86
2023-02-06 01:18:48	37.2147	37.0243	8	6.58	2023-02-06 01:17:33	1.5	73.3	68.7	458	239	86
2023-02-06 01:18:50	37.2147	37.0243	8	6.58	2023-02-06 01:17:33	1.5	75.3	70.7	458	239	86
2023-02-06 01:18:52	37.2147	37.0243	8	6.58	2023-02-06 01:17:33	1.5	77.9	73.3	458	239	86
2023-02-06 01:18:55	37.2147	37.0243	8	6.58	2023-02-06 01:17:33	1.5	80.2	75.6	458	239	86
2023-02-06 01:18:56	37.2147	37.0243	8	6.58	2023-02-06 01:17:33	1.5	82.0	77.4	458	239	86

Table S1: EPIC alerts for the Pazarcik earthquake. These correspond to the alert progression plotted in Figure 2a. Location errors are calculated with respect to the USGS epicenter. Alert radii for alert thresholds of MMI 3, 4, and 5 are calculated using the approach outlined in the Methods section of the Main Text.

Alert Time (UTC)	EPIC Latitude	EPIC Longitude	EPIC depth (km)	EPIC Magnitude	EPIC Origin Time (UTC)	Location Error (km)	Alert Time after Event Origin (seconds)	Alert Time after First Update (seconds)	MMI3 alert radius (km)	MMI4 Alert Radius (km)	MMI5 Alert Radius (km)
2023-02-06 10:24:59	37.9988	37.4897	8	6.94	2023-02-06 10:24:47	25.8	10.4	0.0	523	291	115
2023-02-06 10:25:01	38.0165	37.2615	8	6.31	2023-02-06 10:24:46	5.8	12.4	1.9	407	200	66
2023-02-06 10:25:03	38.0165	37.2615	8	6.68	2023-02-06 10:24:46	5.8	14.1	3.7	477	254	94
2023-02-06 10:25:06	38.0165	37.2615	8	6.73	2023-02-06 10:24:46	5.8	16.7	6.3	486	261	98
2023-02-06 10:25:08	38.0165	37.2615	8	6.79	2023-02-06 10:24:46	5.8	18.8	8.4	497	270	103
2023-02-06 10:25:10	38.0165	37.2615	8	6.84	2023-02-06 10:24:46	5.8	21.2	10.7	506	277	107
2023-02-06 10:25:12	38.0165	37.2615	8	6.89	2023-02-06 10:24:46	5.8	23.1	12.7	515	284	111
2023-02-06 10:25:14	38.0165	37.2615	8	6.91	2023-02-06 10:24:46	5.8	25.2	14.7	518	287	112
2023-02-06 10:25:16	38.0165	37.2615	8	7.02	2023-02-06 10:24:46	5.8	27.4	16.9	538	302	122
2023-02-06 10:25:18	38.0165	37.2615	8	7.13	2023-02-06 10:24:46	5.8	29.2	18.8	557	319	132
2023-02-06 10:25:20	38.0165	37.2615	8	7.21	2023-02-06 10:24:46	5.8	31.3	20.9	571	330	140
2023-02-06 10:25:22	38.0165	37.2615	8	7.26	2023-02-06 10:24:46	5.8	33.3	22.8	580	338	144
2023-02-06 10:25:24	38.0165	37.2615	8	7.29	2023-02-06 10:24:46	5.8	35.3	24.9	584	342	147
2023-02-06 10:25:26	38.0165	37.2615	8	7.29	2023-02-06 10:24:46	5.8	37.4	27.0	584	342	147
2023-02-06 10:25:28	38.0165	37.2615	8	7.30	2023-02-06 10:24:46	5.8	39.3	28.9	586	343	148
2023-02-06 10:25:30	38.0165	37.2615	8	7.30	2023-02-06 10:24:46	5.8	41.2	30.8	586	343	148
2023-02-06 10:25:32	38.0165	37.2615	8	7.30	2023-02-06 10:24:46	5.8	43.3	32.9	586	343	148
2023-02-06 10:25:34	38.0165	37.2615	8	7.30	2023-02-06 10:24:46	5.8	45.4	35.0	586	343	148
2023-02-06 10:25:36	38.0165	37.2615	8	7.30	2023-02-06 10:24:46	5.8	47.5	37.0	586	343	148
2023-02-06 10:25:38	38.0165	37.2615	8	7.30	2023-02-06 10:24:46	5.8	49.3	38.9	586	343	148
2023-02-06 10:25:40	38.0165	37.2615	8	7.30	2023-02-06 10:24:46	5.8	51.5	41.0	586	343	148
2023-02-06 10:25:42	38.0165	37.2615	8	7.30	2023-02-06 10:24:46	5.8	53.5	43.0	586	343	148
2023-02-06 10:25:44	38.0165	37.2615	8	7.30	2023-02-06 10:24:46	5.8	55.3	44.9	586	343	148
2023-02-06 10:25:46	38.0165	37.2615	8	7.30	2023-02-06 10:24:46	5.8	57.4	47.0	586	343	148
2023-02-06 10:25:48	38.0165	37.2615	8	7.30	2023-02-06 10:24:46	5.8	59.3	48.9	586	343	148
2023-02-06 10:25:50	38.0165	37.2615	8	7.30	2023-02-06 10:24:46	5.8	61.6	51.2	586	343	148
2023-02-06 10:25:52	38.0165	37.2615	8	7.30	2023-02-06 10:24:46	5.8	63.7	53.2	586	343	148
2023-02-06 10:25:55	38.0165	37.2615	8	7.30	2023-02-06 10:24:46	5.8	65.7	55.3	586	343	148
2023-02-06 10:25:57	38.0165	37.2615	8	7.30	2023-02-06 10:24:46	5.8	68.0	57.6	586	343	148
2023-02-06 10:25:59	38.0165	37.2615	8	7.30	2023-02-06 10:24:46	5.8	70.0	59.6	586	343	148
2023-02-06 10:26:01	38.0165	37.2615	8	7.30	2023-02-06 10:24:46	5.8	71.8	61.3	586	343	148
2023-02-06 10:26:03	38.0165	37.2615	8	7.30	2023-02-06 10:24:46	5.8	74.4	63.9	586	343	148
2023-02-06 10:26:06	38.0165	37.2615	8	7.30	2023-02-06 10:24:46	5.8	76.8	66.4	586	343	148
2023-02-06 10:26:08	38.0165	37.2615	8	7.30	2023-02-06 10:24:46	5.8	79.3	68.8	586	343	148
2023-02-06 10:26:10	38.0165	37.2615	8	7.30	2023-02-06 10:24:46	5.8	81.0	70.5	586	343	148

Table S2: EPIC alerts for the Elbistan earthquake. These correspond to the alert progression plotted in Figure 2b. Location errors are calculated with respect to the USGS epicenter. Alert radii for alert thresholds of MMI 3, 4, and 5 are calculated using the approach outlined in the Methods section of the Main Text.

Alert Time (UTC)	EPIC Latitude	EPIC Longitude	EPIC depth (km)	EPIC Magnitude	EPIC Origin Time (UTC)	Location Error (km)	Alert Time after Event Origin (seconds)	Alert Time after First Update (seconds)	MMI3 alert radius (km)	MMI4 Alert Radius (km)	MMI5 Alert Radius (km)
2023-02-20 17:04:34	36.1326	36.1477	8	5.90	2023-02-20 17:04:30	11.5	4.0	0.0	329	145	42
2023-02-20 17:04:36	36.1506	36.1477	8	6.53	2023-02-20 17:04:30	11.1	5.8	1.8	448	231	82
2023-02-20 17:04:37	36.2585	36.0808	8	6.54	2023-02-20 17:04:29	11.9	7.5	3.5	450	233	82
2023-02-20 17:04:40	36.2585	36.0808	8	6.48	2023-02-20 17:04:29	11.9	10.2	6.2	439	224	78
2023-02-20 17:04:42	36.2585	36.0808	8	6.43	2023-02-20 17:04:29	11.9	12.1	8.1	430	217	74
2023-02-20 17:04:43	36.2585	36.0808	8	6.42	2023-02-20 17:04:29	11.9	13.6	9.6	428	216	73
2023-02-20 17:04:46	36.2585	36.0808	8	6.34	2023-02-20 17:04:29	11.9	15.9	11.9	412	204	68
2023-02-20 17:04:50	36.2585	36.0808	8	6.31	2023-02-20 17:04:29	11.9	20.1	16.1	407	200	66
2023-02-20 17:04:52	36.2585	36.0808	8	6.31	2023-02-20 17:04:29	11.9	22.4	18.4	407	200	66
2023-02-20 17:04:54	36.2585	36.0808	8	6.29	2023-02-20 17:04:29	11.9	24.3	20.4	403	197	65
2023-02-20 17:04:56	36.2585	36.0808	8	6.22	2023-02-20 17:04:29	11.9	26.5	22.6	390	188	60
2023-02-20 17:04:58	36.2585	36.0808	8	6.21	2023-02-20 17:04:29	11.9	28.5	24.6	388	186	59
2023-02-20 17:05:00	36.2585	36.0808	8	6.16	2023-02-20 17:04:29	11.9	30.5	26.6	378	179	57
2023-02-20 17:05:02	36.2585	36.0808	8	6.17	2023-02-20 17:04:29	11.9	32.3	28.4	380	180	57
2023-02-20 17:05:04	36.2585	36.0808	8	6.15	2023-02-20 17:04:29	11.9	34.7	30.7	376	177	56
2023-02-20 17:05:06	36.2585	36.0808	8	6.16	2023-02-20 17:04:29	11.9	36.0	32.1	378	179	57
2023-02-20 17:05:08	36.2585	36.0808	8	6.16	2023-02-20 17:04:29	11.9	38.6	34.7	378	179	57
2023-02-20 17:05:11	36.2585	36.0808	8	6.15	2023-02-20 17:04:29	11.9	40.8	36.8	376	177	56
2023-02-20 17:05:13	36.2585	36.0808	8	6.15	2023-02-20 17:04:29	11.9	43.4	39.5	376	177	56
2023-02-20 17:05:15	36.2585	36.0808	8	6.15	2023-02-20 17:04:29	11.9	45.3	41.3	376	177	56
2023-02-20 17:05:17	36.2585	36.0808	8	6.15	2023-02-20 17:04:29	11.9	47.5	43.6	376	177	56
2023-02-20 17:05:20	36.2585	36.0808	8	6.15	2023-02-20 17:04:29	11.9	50.5	46.5	376	177	56
2023-02-20 17:05:22	36.2585	36.0808	8	6.15	2023-02-20 17:04:29	11.9	52.3	48.3	376	177	56
2023-02-20 17:05:25	36.2585	36.0808	8	6.15	2023-02-20 17:04:29	11.9	55.3	51.4	376	177	56
2023-02-20 17:05:27	36.2585	36.0808	8	6.15	2023-02-20 17:04:29	11.9	57.6	53.6	376	177	56
2023-02-20 17:05:29	36.2585	36.0808	8	6.15	2023-02-20 17:04:29	11.9	59.4	55.4	376	177	56
2023-02-20 17:05:31	36.2585	36.0808	8	6.15	2023-02-20 17:04:29	11.9	61.5	57.5	376	177	56
2023-02-20 17:05:33	36.2585	36.0808	8	6.15	2023-02-20 17:04:29	11.9	63.7	59.7	376	177	56
2023-02-20 17:05:36	36.2585	36.0808	8	6.15	2023-02-20 17:04:29	11.9	66.2	62.3	376	177	56
2023-02-20 17:05:38	36.2585	36.0808	8	6.15	2023-02-20 17:04:29	11.9	68.5	64.5	376	177	56
2023-02-20 17:05:41	36.2585	36.0808	8	6.15	2023-02-20 17:04:29	11.9	71.0	67.0	376	177	56
2023-02-20 17:05:43	36.2585	36.0808	8	6.15	2023-02-20 17:04:29	11.9	73.7	69.7	376	177	56
2023-02-20 17:05:46	36.2585	36.0808	8	6.15	2023-02-20 17:04:29	11.9	76.3	72.3	376	177	56
2023-02-06 01:18:48	37.2147	37.0243	8	6.58	2023-02-06 01:17:33	1.5	73.3	68.7	458	239	86
2023-02-06 01:18:50	37.2147	37.0243	8	6.58	2023-02-06 01:17:33	1.5	75.3	70.7	458	239	86
2023-02-06 01:18:52	37.2147	37.0243	8	6.58	2023-02-06 01:17:33	1.5	77.9	73.3	458	239	86
2023-02-06 01:18:55	37.2147	37.0243	8	6.58	2023-02-06 01:17:33	1.5	80.2	75.6	458	239	86
2023-02-06 01:18:56	37.2147	37.0243	8	6.58	2023-02-06 01:17:33	1.5	82.0	77.4	458	239	86

Table S3: EPIC alerts for the Yayladagi earthquake. These correspond to the alert progression plotted in Figure 2c. Location errors are calculated with respect to the USGS epicenter. Alert radii for alert thresholds of MMI 3, 4, and 5 are calculated using the approach outlined in the Methods section of the Main Text.

Source parameters and EPIC Magnitude Estimates

In large earthquakes, ground motion amplitudes may be influenced by source properties such as directivity, stress drop, rupture velocity etc., as well as other factors arising from path and site effects. Finite fault models (FFMs)[9–11] have revealed the two mainshocks of the Kahranmanmaraş sequence involved bilateral rupture, with a complex source evolution and kinematic rupture characteristics, such as variable rupture velocities on different rupture segments. As EPIC uses highpass-filtered peak-ground displacement from single stations in a narrow time window to estimate earthquake magnitude in real-time, we seek here to quantify how source characteristics can influence EPIC magnitude estimates. For example, we would expect unilateral rupture directivity to manifest as increased ground motion amplitudes and reduced apparent durations in the forward directivity direction. Larger rupture velocities create larger differences between the forward and backward directivity directions[12]. We investigate here whether source properties, site effects, or a combination of both, might explain any patterns seen in individual station magnitudes (M_{Pd}) and measured peak P-wave amplitude in the EPIC 4-second window (P_d).

We first look at individual station P_d magnitudes in the Pazarcık event in Figure S5. FFMs of the Pazarcık sequence showed bilateral rupture with a similar, subshear rupture velocity on both segments, although some studies[13] have raised the possibility for localized supershear rupture. We would not expect significant differences in station magnitudes due to directivity, given that these rupture differences were not seen until later in the rupture, outside of EPIC's 4-second window. The Goldberg et al.[10] FFM indicates slip predominantly propagating on the minor Narlı segment towards the NE, which might explain the high station magnitudes at stations TK.4615 and KO.KHMN (see Figure S1b), located to the NE of the epicenter. High station magnitudes in the station cluster in the town of Kahranmanmaraş (azimuth of 340-350 degrees), might also be related to this.

The data for this event does not seem to show a clear-cut relationship between station magnitude and site effects. The shear-wave velocity in the top 30 m (V_{S30}) is commonly used as an engineering proxy for site amplification. A map mosaic of V_{S30} in the region is available, and a number of TK stations have measured V_{S30} values. If site effects were dominant, we would expect to see a general trend of low V_{S30} correlating with high P_d amplitudes in Figure S5d. Instead, there is no clear relationship and significant scatter. Some of the stations with the lowest M_{Pd} estimates are found along the East Anatolian fault, in areas of moderate V_{S30} (see Figure S5c).

We next look for a potential source effect in the EPIC station magnitudes in the Elbistan event. Both Jia et al.[11] and Melgar et al.[9] closely investigated how varying rupture velocity improved FFM fit to the strong motion and high-rate geodetic displacement data for different rupture segments. They both concluded that rupture in the event was bilateral, but found that the much higher displacement amplitudes towards the west required supershear rupture on the westward segment (with a rupture velocity, V_R, of 4.5–4.8 km/s), while the eastward segment could be fit with a subshear V_R of 2.5–2.8 km/s. These large differences in rupture velocity were required from the start of the rupture, so they could have influenced ground motion amplitudes in the 4-second EPIC window. Figure S6b shows that station M_{Pd} values to the west of the epicenter (azimuths 270-300 degrees), in the supershear rupture direction, are indeed somewhat higher than those to the east. Like in the case of the Pazarcık event, panels c and d, show a scattered relationship between M_{Pd}/P_d and V_{S30}.

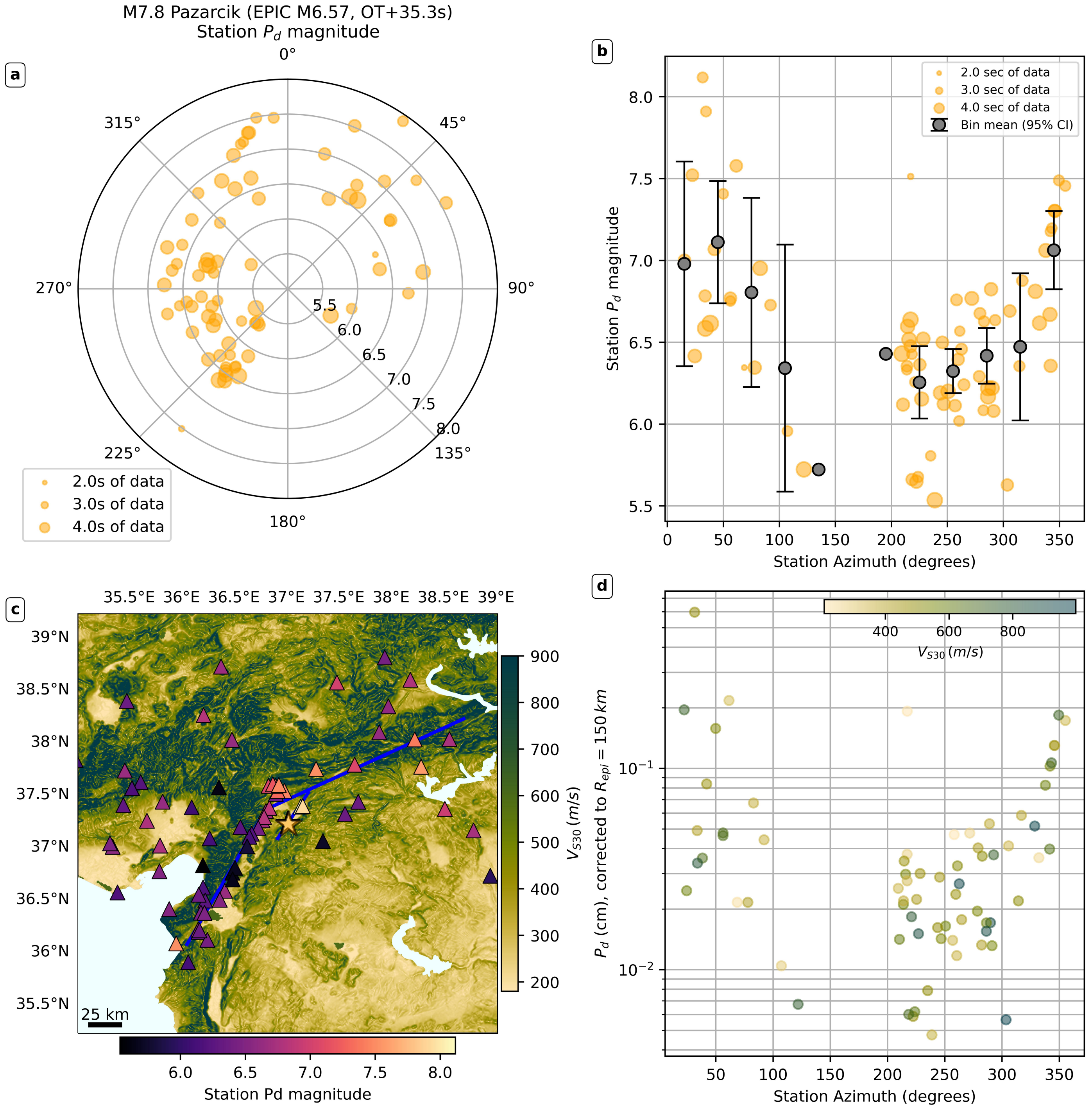

Figure S5: Directivity in EPIC station P_d magnitudes for the $M7.8$ Pazarcık event. We plot the station magnitude estimates for an update at 36 seconds from OT, for which the aggregated, weighted average magnitude estimate was 6.58. a) Polar plot of station P_d magnitudes, plotted against azimuth from the epicenter. Each station is represented by one dot, scaled according to the amount of data cotntributed by each station. b) Station P_d magnitudes and binned means, against station azimuth. Error bars show the 95% confidence interval (CI) of the binned mean. b) Spatial distribution of stations contributing M_{Pd} for this update, plotted on top of V_{S30} estimates. The V_{S30} grid is sourced from the USGS global mosaic[14]. Each station is plotted as a triangle and colored by its M_{Pd} value. c) Plot of station peak P-wave amplitude, P_d, against azimuth. We correct station P_d to a common distance of 150 km using the distance scaling in the KA13 scaling law. Each station is represented by a dot, colored by the station V_{S30} (taken from the AFAD inventory).

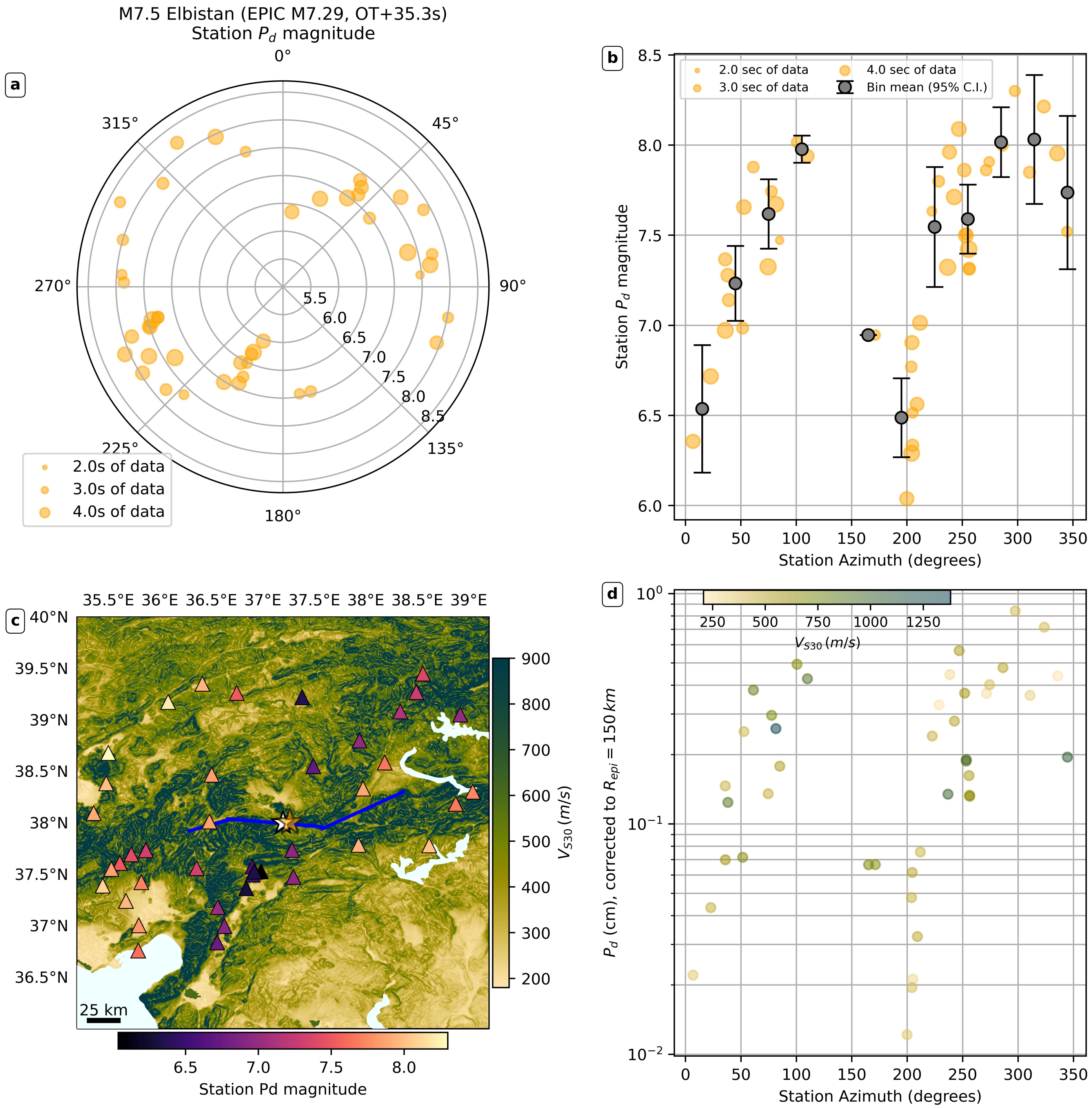

Figure S6: As in Figure S5, but the $M7.5$ Elbistan event.

MyShake Alert Processing Workflow

MyShake is a citizen science smartphone app, available globally for Android and iOS smartphones. For users in the US West Coast states of California, Oregon, and Washington, MyShake acts as a delivery partner for ShakeAlert early warning messages. It has been delivering alerts to users in California since October 2019, in Oregon since March 2021, and in Washington since January 2022[15,16]. The current MyShake processing workflow is described below, modified from previous work[15,16].

MyShake alerts users based on the predicted extent of MMI 3 shaking included in the contour product sent out by the ShakeAlert alert layer[1,15,17]. The alert processing workflow is triggered by receipt of a ShakeAlert alert initial update for an earthquake event. Using the octagon contour product for MMI 3 shaking included in the ShakeAlert update XML, the MyShake backend calculates which devices are within the current alert region. Phones are grouped into Military Grid Reference System (MGRS) cells with a 10x10 km resolution. Users are assigned to an MGRS cell based on a preferred alerting location (called Homebase), or the latest available location (for users who enable location sharing permissions). These lists of users in each MGRS cell are stored in an alerting cache.

MyShake determines which MGRS cells to alert by calculating which cells lie within or intersect the ShakeAlert alert contour polygon. To further speed up processing, users are assigned into parallel processing batches that process incoming alert updates simultaneously. This assignment process is random at the point of app download, such that each batch ends up with a random distribution of users in space. User penetration is monitored to ensure that each batch is not responsible for handling more than 100,000 users at any given time. When this is no longer possible, latency performance is maintained by adding more batches. In turn, this means that there will be more parallel runs (see below for definition) processing each alert update.

Each incoming ShakeAlert update is processed independently for each batch, by a single subprocess, which we term an alerting run. A run will determine the MGRS cells that the alert contour intersects and alert the devices that have been assigned to it, by pushing the list of targeted devices to message distribution services. Once processing of an alert finishes, the backend checks for further updates in the ShakeAlert stream and determines whether the latest update pertains to the same event. This is done by comparing the previous and current epicenters and origin times and ensuring that they do not differ by more than 100 km and 16 s, respectively. If this check is passed, further runs will recalculate the cells that are included in the alert region, and alert users in each batch accordingly. Each cell, and hence each device (each user), only receives one alert for a given event.

To assess delivery performance, the MyShake app on individual devices will send back a timestamped "heartbeat" and further analytics data containing acknowledgement of an alert receipt[16]. Individual receipts can be traced back through the system and associated with the start of processing time. This allows calculation of the time interval from when MyShake received an alert update from ShakeAlert until the phone received the alert, for all users for which a receipt is received[15]. This metric measures the performance of both the MyShake backend as well as the delivery services used in terms of their timeliness.

MyShake Latency Modeling

We sought to develop a statistical model of alert that captures the broad trends in observed MyShake delivery latencies. We draw on real-world latency data from MyShake. First, we introduce the alerting workflow used in MyShake, which is the process that controls the delivery latency.

MyShake Alerting Workflow

MyShake implements parallelized alert delivery (see Methods in the Main Text and MyShake Alert Processing Workflow section above). As such, for each new ShakeAlert alert message received, there is one alerting run, that is, one realization of the stochastic latency generation process, targeting N users. In each event, there may be multiple alert updates (as is the case in our Kahramanmaraş sequence replays), and each update is processed by multiple parallel subprocesses (one for each user batch). Therefore, there are many runs which target users in an event for which MyShake alerts are issued.

MyShake Latency Modeling

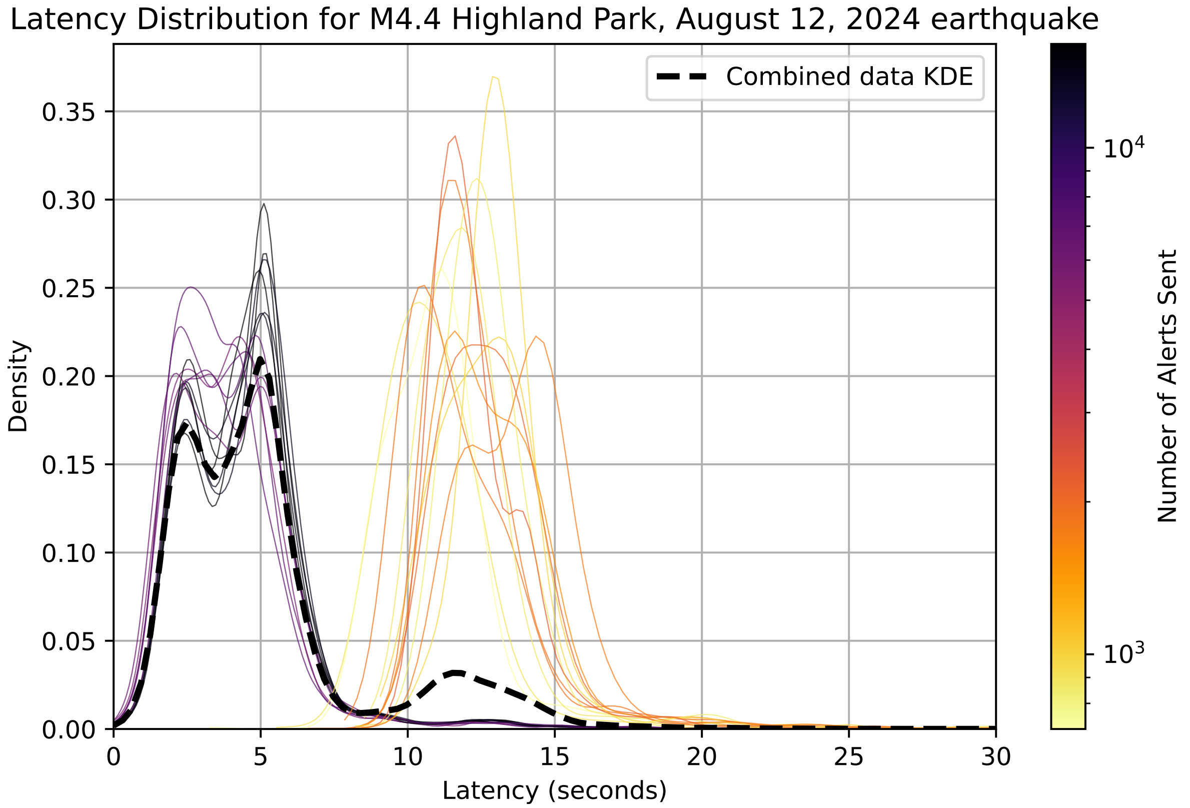

Figure S7: MyShake latency distribution for the August 12, 2024, M4.4 Highland Park earthquake in Los Angeles, for which MyShake sent 381,255 alerts. We plot the overall distribution of latencies using a kernel density estimate (KDE) shown by the dashed black line. Individual lines show KDEs for the latency distributions of individual runs, with each line colored by the number of alerts sent (i.e. the number of users targeted by the run).

Operationally, MyShake uses automatic alert receipts to produce latency statistics for each alerting run[15,16]. Patel and Allen[15] and Marcou et al.[16] reported positively skewed alert delivery time distributions using data from moderate earthquakes, an observation that has remained true even as the system has processed larger and larger events in terms of alerts sent[16]. For our modeling, we use real-world alert delivery data from all events

processed by MyShake since the 2019 launch of public alerting, where 10,000 or more alerts were sent (total of over 1.4 million raw alert receipts) to develop our latency model.

Looking across events, and specifically at the latency medians across individual runs (Figure 3 in the Main Text), we observed an increasing linear trend in median latency in log-space with the number of alerts sent, albeit with a large scatter.

Looking at a single example event in Figure S7, the M4.4 Highland Park, Los Angeles earthquake on August 12, 2024 (almost MyShake 400,000 alerts sent), gives a good representation of first order trends of latency distribution shape. The overall latency distribution for the event, shown by the dashed black line, is a weighted average of the latency distributions of individual MyShake alerting runs (shown by the colored lines, total of 24 completed runs that sent alerts for this event). Runs that send more alerts (e.g. the dark colored runs), weigh more in the overall latency distribution shape. In contrast to the usual trend (shown by Figure 3 in the Main Text), runs that sent fewer alerts seem to have higher latencies in this particular earthquake; this is an anomaly. We can also observe that individual runs share two common characteristics, despite large variability in the exact distribution shape: they are generally right- (i.e., positively) skewed, and have long tails.

We therefore decided to separately develop models for latency medians and the shape of the latency distributions. For both steps, we used Bayesian parametric statistical modeling techniques in order to get explicit uncertainty estimates for the values of estimated parameters.

Under Bayesian inference, Bayes' rule is used to infer the most likely model, θ, with an associated set of hyperparameters α, given the set of data points, $\mathbf{X}$. In Bayesian inference, the probability of the model given the data and a set of hyperparameters, $p(\theta|\mathbf{X}, \alpha)$ (the posterior) is often approximated by:

$$p(\theta|\mathbf{X}, \alpha) \propto \; p(\theta|\alpha)p(\mathbf{X}, \alpha|\theta) \tag{1}$$

where $p(\theta|\alpha)$ is the prior belief about the model, and $p(\mathbf{X}, \alpha|\theta)$ is the likelihood function.

Latency Medians

We build our latency median model by considering 50th percentile (median) delivery latencies for individual runs in events for which a total of 10,000 or more alerts were sent, and for which 100 or more individual latency observations were available (total of 501 runs). We observed a general increase of alert delivery latency with increasing number of devices targeted by a given run (Figure 3 in the Main Text). This intuitively makes sense, as more alerts sent by a particular run mean a greater system load.

We proceeded to model the medians in a Bayesian framework, whereby:

$$t_{delivery,median} \sim N(t_{model}, \sigma_N) \tag{2}$$

where t_{model} is given by the linear model:

$$log_{10}(t_{model}) = a + b\ log_{10}(n_{sent}) \tag{3}$$

We use the weakly informative priors in Table S4. We sample 1,000 samples from priors using the No-U-Turn Sampler (NUTS)[18] using 4 concurrent chains, and 1,000 burn-in samples to eliminate any potential bias from the algorithm's choice of starting point for sampling.

Parameter	Priors	Posteriors (mean and standard deviation)
a (intercept)	$a \sim N(\mu = 0, \sigma = 1)$	$a = -0.109 \pm 0.0.060$
b (slope)	$b \sim N(\mu = 0, \sigma = 5)$	$b = 0.117 \pm 0.017$
σ_N (noise)	$\sigma_N \sim HalfN(\sigma = 5)$	$\sigma_N = 0.215 \pm 0.007$

Table S4: Priors and posteriors for the linear model parameters.

Our posterior parameter estimates are tight, even though metrics showed that the parameter space was explored well. Our final mean model for the median latency was:

$$log_{10}(t_{model}) = -0.109 + 0.177\ log_{10}(n_{alerts})$$

Latency Distribution Shape

We sought to produce one parametric model for the shape of the latency distribution, in order to capture the broad characteristics of latency distributions: the right-skewness and long tails. These characteristics seem to repeat across events. There is significant inter-event variability in the exact shapes (see Figure S8), including evidence for second-order features like bi-modality. We chose to develop a relatively simple model, and therefore ignored second-order features.

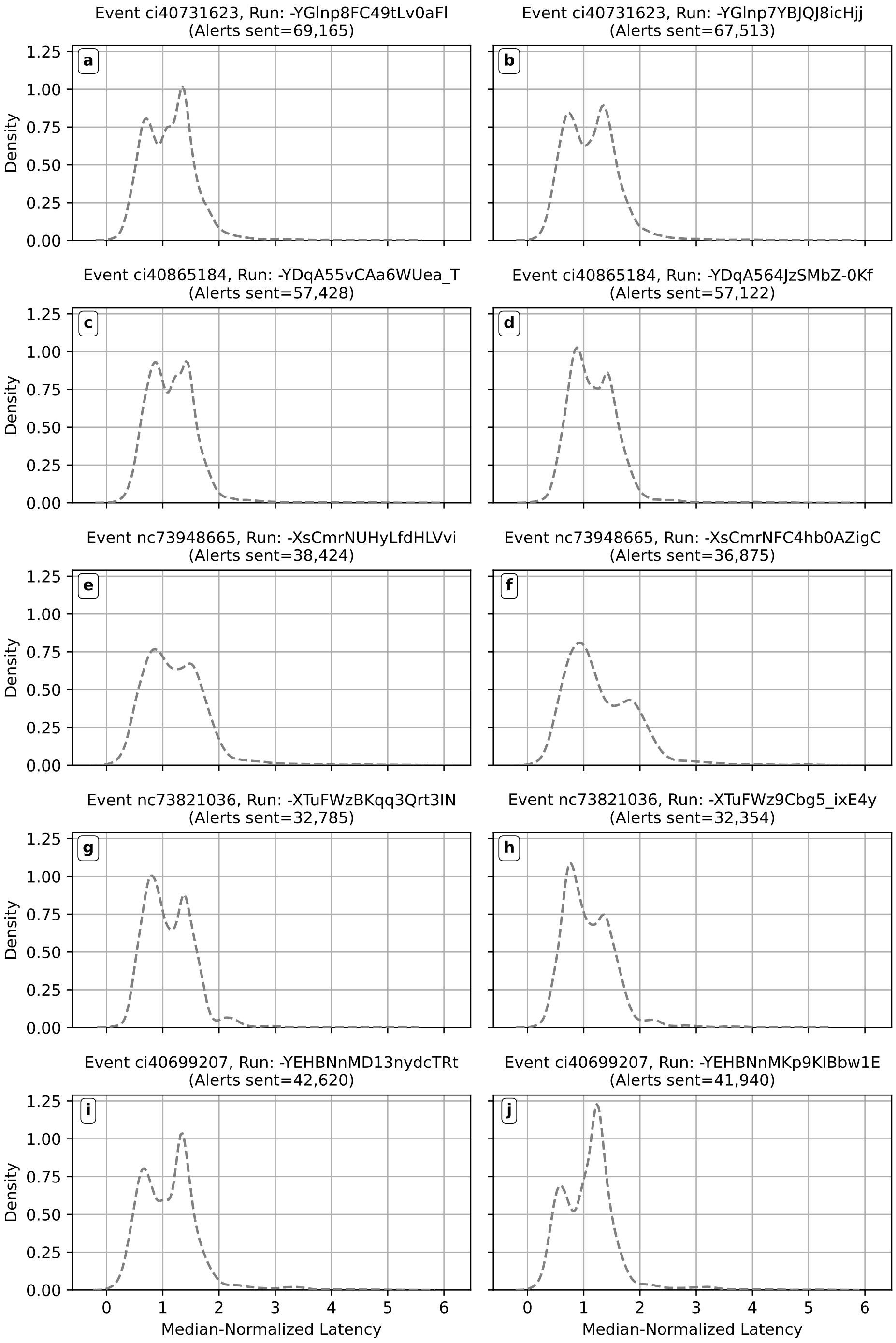

Figure S8: Shapes of latency distributions for individual runs across 5 events for which MyShake sent large numbers of alerts. Each panel shows the event ComCat ID and the number of alerts sent by that run. For each run, we plot a KDE of the latency distribution, shown as a dashed grey line. The x-axis represents normalized latency. For each run, we normalize latency by dividing by the run median latency.

We therefore built our dataset for the latency shape modeling by aggregating over 1.4 million individual delivery latency observations across 806 distinct runs, involving 33 events between 2020 and the end of 2024 for which MyShake sent a total of 10,000 or more alerts. We create our aggregated dataset by first non-dimensionalizing latency observations by dividing each data point by the median latency of the alerting run to which it belonged. We thus create observations that focus exclusively on the shape of the distribution. Given the non-dimensionalized latency data (now expressing normalized latency, as a multiple of the median), we created a single unified dataset for shape modeling. By aggregating the data into a single dataset, we effectively allowed runs with more receipts a higher weight in determining the overall, aggregate latency distribution shape.

We tested four candidate distributions: a Weibull model, a Gamma model, a lognormal model, and a loglogistic (Fisk) model. All candidate distributions are in principle able to produce the broad characteristics we observe, and additionally, ensure all delivery latencies are positive.

Model	Parameter priors	Parameter posterior (means)	$ELPD_{WAIC}$	SE ELPD	p_{WAIC}	D_{KL}
**Weibull**	$\lambda \sim Gamma(\alpha = 1.0, \beta = 1.0)$<br>$\kappa \sim Gamma(\alpha = 1.0, \beta = 1.0)$	$\lambda = 2.205 \pm 0.004$ (s.d.)<br>$\kappa = 1.191 \pm 0.002$ (s.d.)	-94125.4	446.881	5.737	0.5796
**Gamma**	$\alpha \sim Gamma(\alpha = 1.0, \beta = 1.0)$<br>$\beta \sim Gamma(\alpha = 1.0, \beta = 1.0)$	$\alpha = 5.189 \pm 0.019$ (s.d.)<br>$\beta = 4.916 \pm 0.019$ (s.d.)	-84393.3	398.739	3.157	0.0340
**Loglogistic***	$mu \sim Normal(\mu = 0.0, \sigma = 10.0)$<br>$s \sim HalfNormal(\sigma = 5.0)$	$mu = -0.029 \pm 0.001$ (s.d.)<br>$s = 0.247 \pm 0.001$ (s.d.)	-88026.3	356.327	2.127	0.0130
**Lognormal**	$\mu \sim Normal(\mu = 0.0, \sigma = 10.0)$<br>$\sigma \sim HalfNormal(\sigma = 10.0)$	$\mu = -0.046 \pm 0.001$ (s.d.)<br>$s = 0.46 \pm 0.001$ (s.d.)	-86628.3	504.692	4.863	0.0447

Table S5: Bayesian modeling setup and results for the latency shape distributions. $ELPD_{WAIC}$ is the expected log pointwise predictive density, calculated using the Watanabe-Akaike Information Criterion (WAIC). SE is the standard error of the $ELPD_{WAIC}$. D_{KL} is the Kullback-Leibler divergence. p_{WAIC} is a measure of model complexity. Note that for the loglogistic case, we modeled the logistic fit to the natural log of the observed data for computational implementation purposes, and then transformed to the loglogistic form. As such the priors and posteriors define parameters for the logistic distribution. These convert to a mean location parameter of $\exp(mu) = 0.971$ and a mean scale parameter of $1/s = 4.049$.

We find best-fitting parameters for these distributions using Bayesian modeling, which also gives explicit estimates of uncertainty in the parameters. We initialized our Bayesian parameter search using weakly - informative priors (Table S5). We searched the parameter space by sampling 2,000 samples from the priors using the No-U-Turn Sampler (NUTS)[18] algorithm using 4 concurrent chains, for a total of 8,000 samples. For each chain, we sampled an additional 1,000 burn-in samples (i.e., discarded from the posterior), to eliminate any potential bias from the algorithm's choice of starting point for sampling.

Our parameter estimates converged to tight posterior samples, as the small posterior standard deviations in Table S5 show. We quantified the fit to the observed distribution using two metrics. First, we used metric $ELPD_{WAIC}$, the expected log-pointwise predictive density (ELPD), calculated using the Watanabe-Akaike Information Criterion (WAIC), which is a metric that quantifies the ability of a model to predict the distribution of a dataset, including a penalty term for model complexity[19,20]. A higher (i.e., less negative) $ELPD_{WAIC}$ indicates a better fit. Second, we used the Kullback-Leibler divergence (D_{KL}), which is a measure of how different two distributions are. It measures the information loss, when a model distribution Q, is used to approximate a true distribution P. A perfect fit would result in $D_{KL} = 0.0$, and thus a lower D_{KL} value indicates that P and Q are not as far apart. As we can see from Table S5, the Weibull model is clearly inferior compared to the Gamma, Lognormal, and Loglogistic models, as it has a smaller $ELPD_{WAIC}$ and a much bigger D_{KL}. There are very small differences between the last three models when we consider $ELPD_{WAIC}$ and D_{KL}.

In order to choose between the models, we turn to posterior predictive checks (PPCs). PPCs generate new synthetic datasets using the likelihood function $p(x \mid \theta)$, where θ is set by the posterior parameter distributions. We generated 100 posterior predictive samples for each candidate distribution, and then compared it to the observed data (see Figure S9), to check the extent to which the candidate models can reproduce the features of

the observed distribution. From this visual check, we chose the loglogistic distribution, due to its ability to best reproduce the shape of the main peak in the aggregate latency distribution, as well as being able to produce samples long along the tail while also deviating the least from the shape of the observed data distribution.

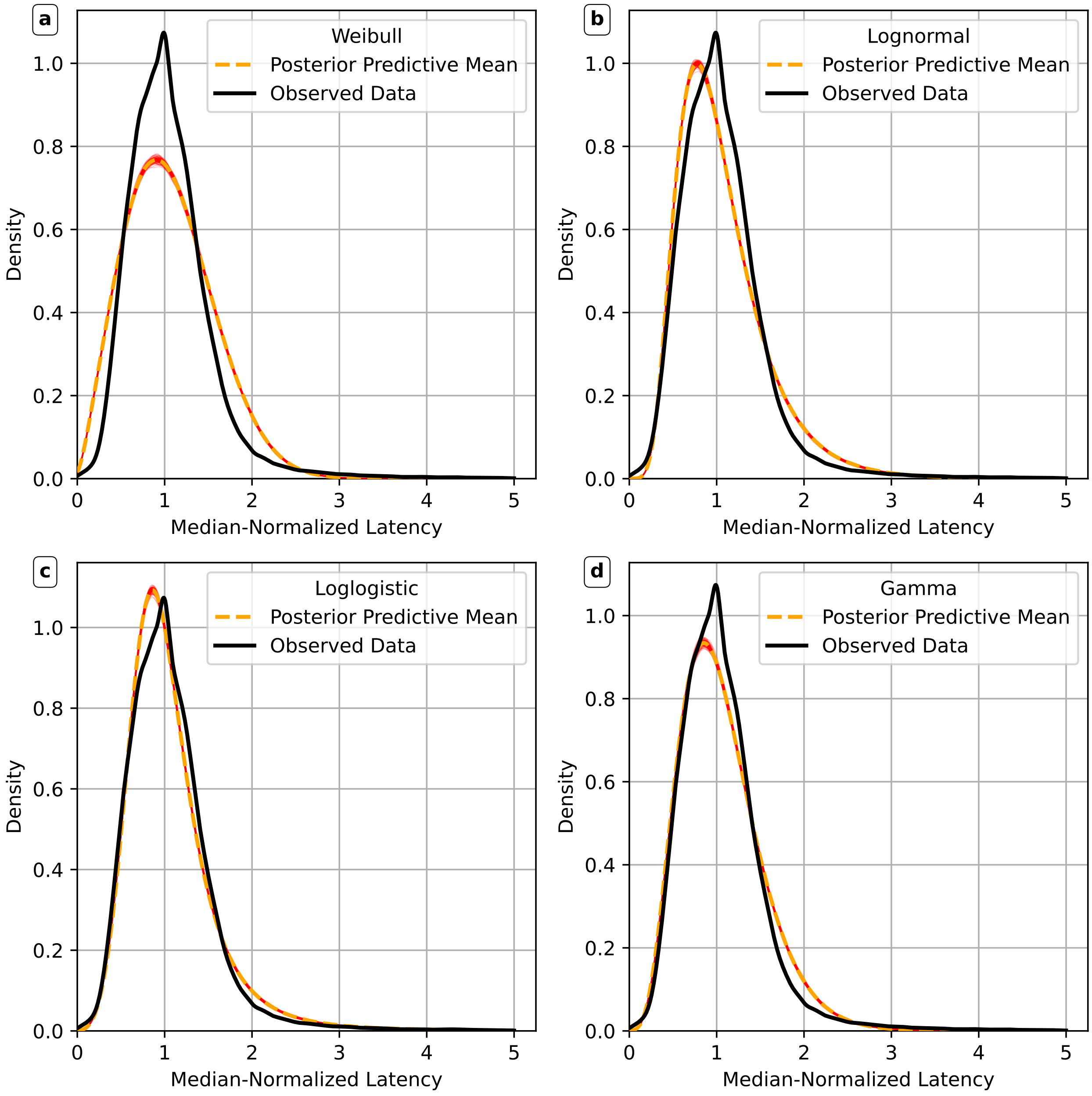

Figure S9: Posterior predictive checks (PPCs) for each of the four candidate models. a) Weibull. b) Gamma. c) Loglogistic. d) Lognormal. The PPC process samples model parameters from the posteriors, and produce simulated datasets using those sampled parameters. Distributions of simulated datasets are then compared to the observed dataset, in this case using kernel density estimates (KDEs). In each panel, we plot 100 posterior predictive samples, the mean KDE for the posterior predictive samples, as well as the KDE of the observed data. The x-axis is plotted out to 5 times the median in all panels.

Warning Time Simulation Workflow

Processing of EPIC Alert Updates for Delivery

Our simulation workflow works by processing updates from EPIC in simulated real time. To simulate the throttling of updates that would occur in a production EEWS, for example as is operational practice in ShakeAlert's Decision Module[1], it checks that any new update is sufficiently different from previous ones before it is used for user alerting. We ensure this by imposing change thresholds of 0.099 units for magnitude and 0.05 degrees in latitude and longitude, similar to how ShakeAlert's solution aggegrator handles updates from the various ShakeAlert algorithms[1]. Updates that exceed these thresholds are passed to a MyShake simulator routine which mimics the real-time MyShake workflow.

Our goal is to simulate warning times for mass public alerting. To simulate user locations, we assumed 10% of the population in the simulation domain has the MyShake app. We then randomly sampled user locations from a 1x1 km population grid[21], following the method of Kong et al.[22,23], who used it to simulate MyShake's earthquake detection and characterization capabilities for smartphone-based EEW. We assigned our sampled user locations in the simulation domain to MGRS cells and user alerting batches. We then ran the simulation with these pre-sampled locations. Recall for each alert update received, MyShake performs one alerting run for every user batch. Each such run calculates which MGRS cells (containing pre-assigned sets of users) interesect the alert polygon (see Figures S10 and S11 for examples of how user locations relate to MGRS cells used for alert targeting). All users that belong to that batch that are found within the intersecting MGRS cells are alerted. Recall that each user is only alerted once, i.e. they are alerted the first time their MGRS is intersected by an alert polygon. For each "run", the time at which EPIC updates are received is recorded as the start of processing (t_{alert} in Equation 1 in the Main Text). Then, the delivery latency distribution for targeted users in each run was simulated using our data-driven statistical model driven by empirical data.

Assigning a Simulated Alert Delivery Latency

For each "run", we set the alert delivery latency as follows. For each update, there are M parallel simulated MyShake runs, each handling a distinct, randomly assigned batch of users. We first calculate the number of users, N, in each batch that need to be alerted. These N users are handled by a single "run", and therefore their alert delivery latency is produced by the same process.

We set the median delivery latency for each run using our linear model based on the number of targeted users. We add noise to this estimate by sampling from the posterior of the noise parameter σ_N, setting up a normal distribution $N \sim (\mu = 0, \sigma = \sigma_N)$, and sampling M samples from this distribution, where M is the number of parallel runs. This noise vector is added to the vector of M median estimates from the scalar model.

We then build a complete latency sample for each of the M runs by drawing N samples from our preferred latency shape distribution, where N is the number of devices targeted for that run. The samples are converted back into physical units by multiplying with the median from the previous step, and are randomly assigned to user locations in the targeted device list for the run.

Calculation of Warning Times via Interpolation

Alert acknowledgment receipts are received discontinuously in space, as they depend on population density (see for example Figure 5a), which in turn governs user density. As such, there does not exist enough density of receipts to simply contour the warning time "field" (see Figure S10 and S11 for examples). The raw warning time dataset has significant scatter, introduced by modeling delivery latency. Additionally, our goal is to jointly illustrate the spatial distribution of shaking intensity and warning time. As seen by comparing the spatial distribution of warning time, and shaking intensity (compare Figure 4a to 4c), shaking intensity shows greater spatial variability than warning time to S-wave arrival, which to first order increases with increasing distance from the epicenter. Note how this trend becomes more apparent once raw user warning times (top row of Figures S10 and S11) are aggregated onto a uniform geographic grid (bottom row of Figures S10 and S11).

We leverage this variation in warning time with distance to produce the warning time contour maps (e.g., Figure 4c-e in the Main Text). We used interpolation methods that are also deployed operationally to measure MyShake and EEW pipeline performance[16]. Given that we measure warning time as the time between alert receipt and arrival of the first S-wave (radiating from the epicenter at a constant moveout velocity of 3.5 km/s), overall trends are captured by modeling the warning times as concentric circles centered on the earthquake epicenter. This is reasonable approximation even though our alert contours are octagonal (to mirror the ShakeAlert contours ingested by MyShake), rather than circular. Alert targeting is done by actually checking which MGRS cells are intersected by the octagonal alert contour, meaning that users within a circular contour of the same radius as the octagonal contour will be fully contained in the alert area (note the full extent of MGRS cells alerted in each case extends past the octagon). The bottom row of Figures S10 and S11 allows for a direct comparison of the interpolated contours and the spatial warning time grid, as well as a view of how the shape of the alerting contours is reflected on the edges of the grid of alerted users.

To do this, we used the full distribution of simulated warning times (see Figure S12, showing MMI3 alerting in the Pazarcik event), mirroring MyShake operational procedure[16]. We calculated the percentiles of warning times in 10 km wide epicentral distance bins and interpolate the bin medians (see gold crosses in Figure S12) in 5 s intervals to calculate the distances at which median warning times occur (see gold squares in Figure S12). This is then converted to a map view plot of warning time contours (e.g. Figure 4b-d) by plotting concentric circles centered at the epicenter with radii matching the interpolated distances. We also interpolated binned 95th latency percentiles to calculate the size of the late-alert zone, by finding the distance at which the monotonically increasing trend line fit through the bin 95th percentiles crosses 0 s warning time (white square in Figure S12). We used the 95th, rather than the 50th percentile, to ensure a warning time that is truly 0 s or less at high confidence (using the median would still mean that 50% of users at the interpolated distance had a positive warning time). Figures S10 and S11, panels d-f, illustrate how our warning time interpolation procedure produces contours that maintain excellent agreement with the first order trends in the spatially discontinuous warning time field. We note that this interpolation method is also used for operational assessment of MyShake's warning time performance - see Marcou et al.[16] for more details and discussion.

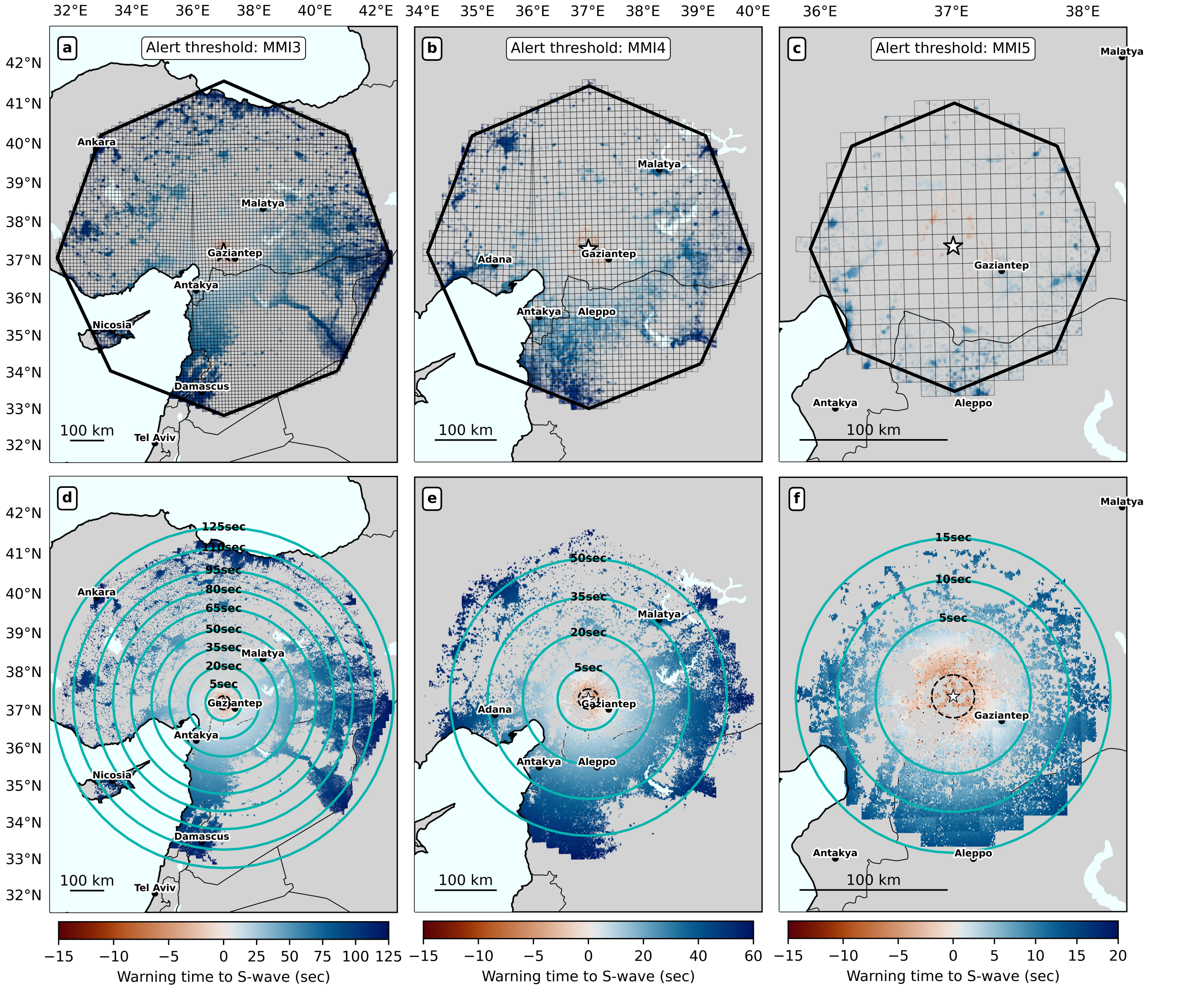

Figure S10: Raw and gridded warning times for the Pazarcik event, across all three alerting thresholds: MMI3 (first column), MMI4 (second column), MMI5 (third column). The top row (panels a, b, c) shows raw, individual user locations, with points colored by individual user warning time. We also show the MGRS geometries used for targeting users for alert delivery in our simulation. The bottom row, show a derived distribution of median gridded warning times on a uniform geographic grid with approximately 1 km resolution (30 arcseconds). Note this is identical to the grids in Figure 4 and 6 in the Main Text. We also show warning time contours from our interpolation workflow. Note how the contours, produced by distance interpolation, capture first order trends in the extremely discontinuous spatial field very well, and how the late-alert zone (dashed black circle) does not seem to match. This is because it is estimated from 95th percentiles, rather than the median. Each panel also shows the catalog epicenter from USGS ComCat as a white star. Note how the panels have different geographic extents.

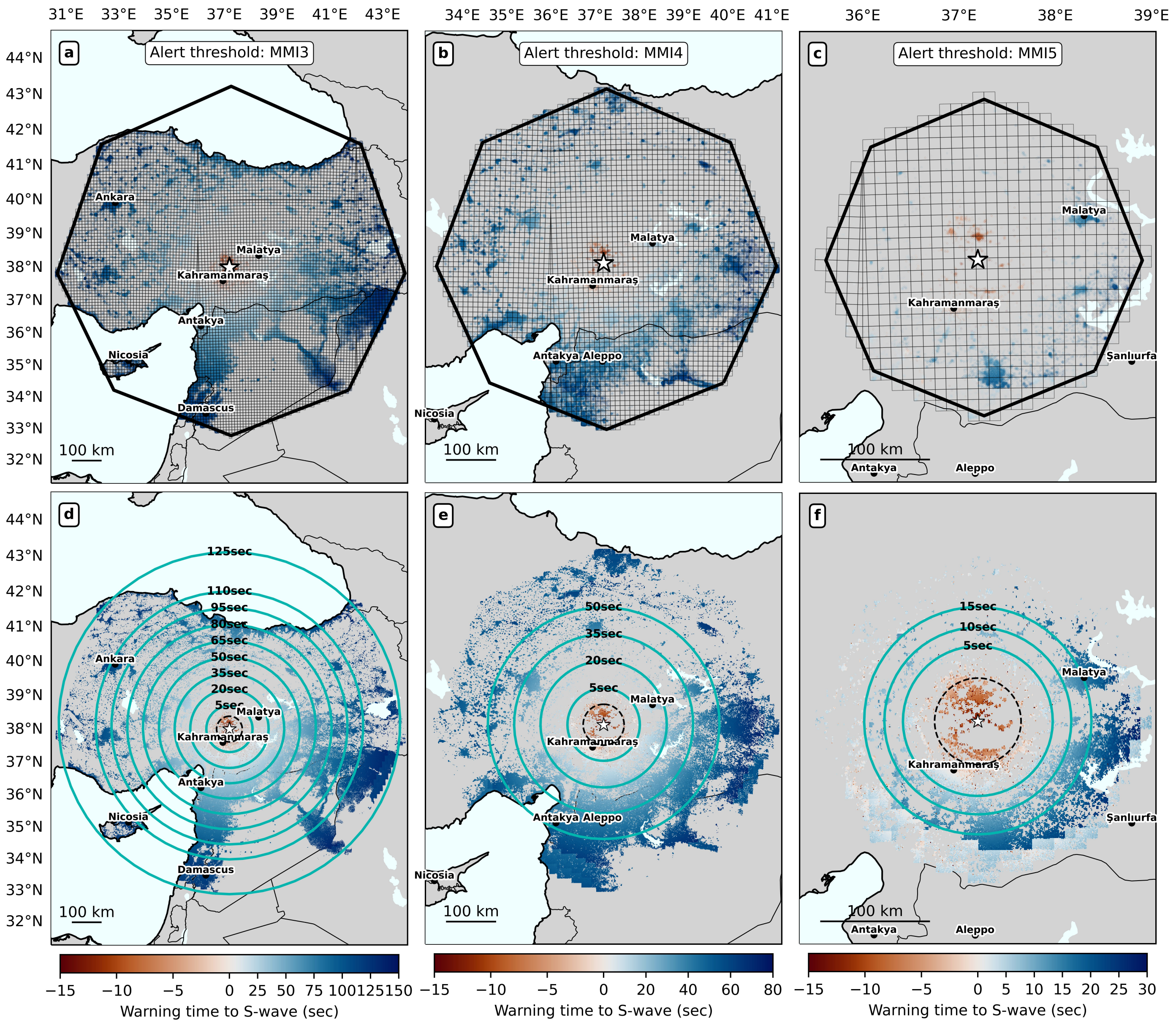

Figure S11: Raw and gridded warning times for the Elbistan event, across all three alerting thresholds: MMI3 (first column), MMI4 (second column), MMI5 (third column). Figure follows an identical structure to Figure S10. Note again how panels in each column have differing geographic extents.

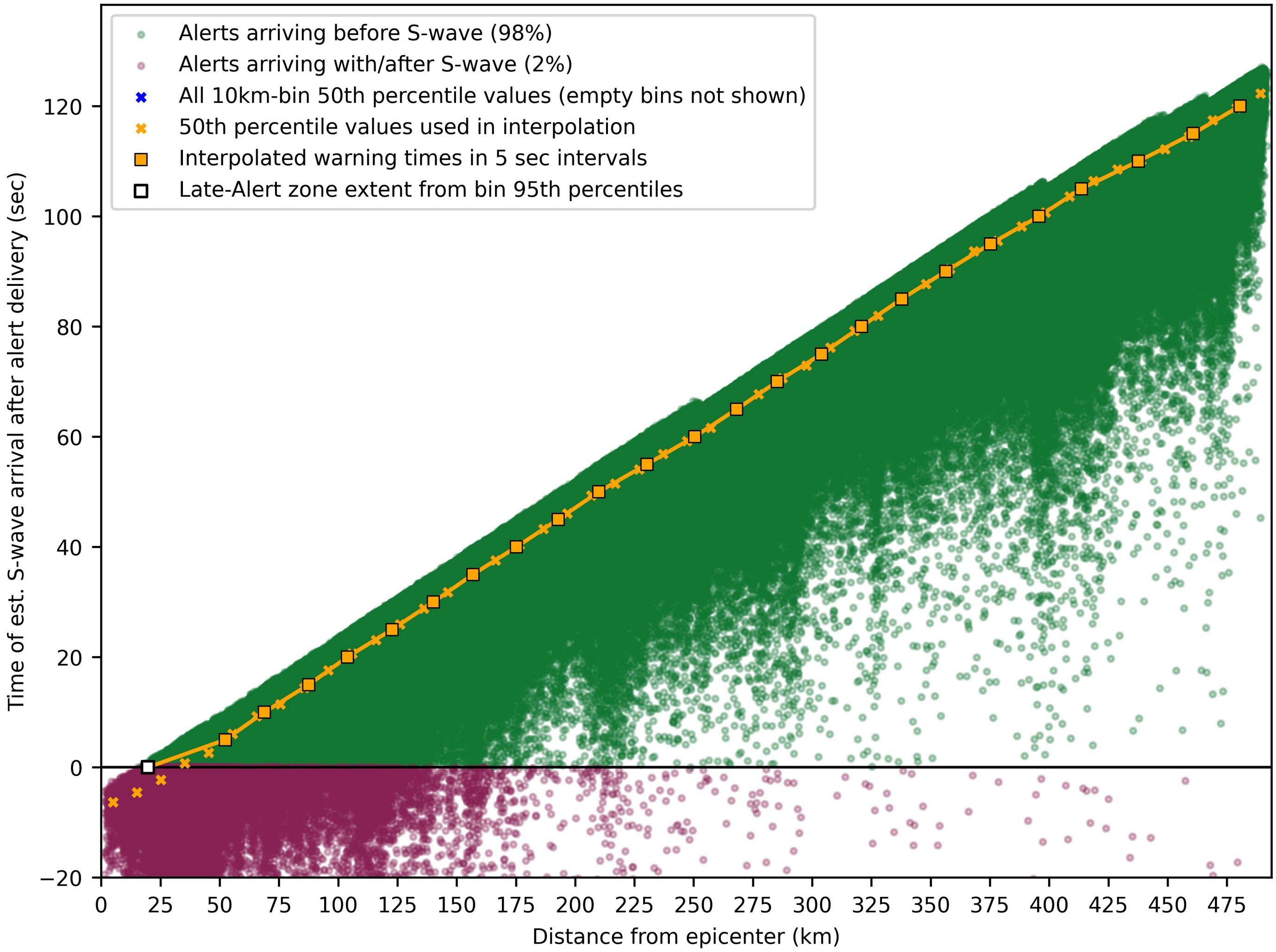

Figure S12: Interpolation process for warning time contours for the M7.8 Pazarcık earthquake. The warning time relative to epicentral distance of the user to the USGS epicenter for the M7.8 Pazarcık earthquake is shown as dots, and colored by whether it was positive (green) or negative (purple). The median warning times for 10 km-wide epicentral distance bins are shown as crosses. Interpolated warning times are shown as golden squares. The size of the blind (no-alert zone) is shown as a black square and is estimated from the interpolation of the bin 95th percentiles, to ensure that the estimate is truly close to the no-alert distance.

Alert Timeliness Analysis, without Latency

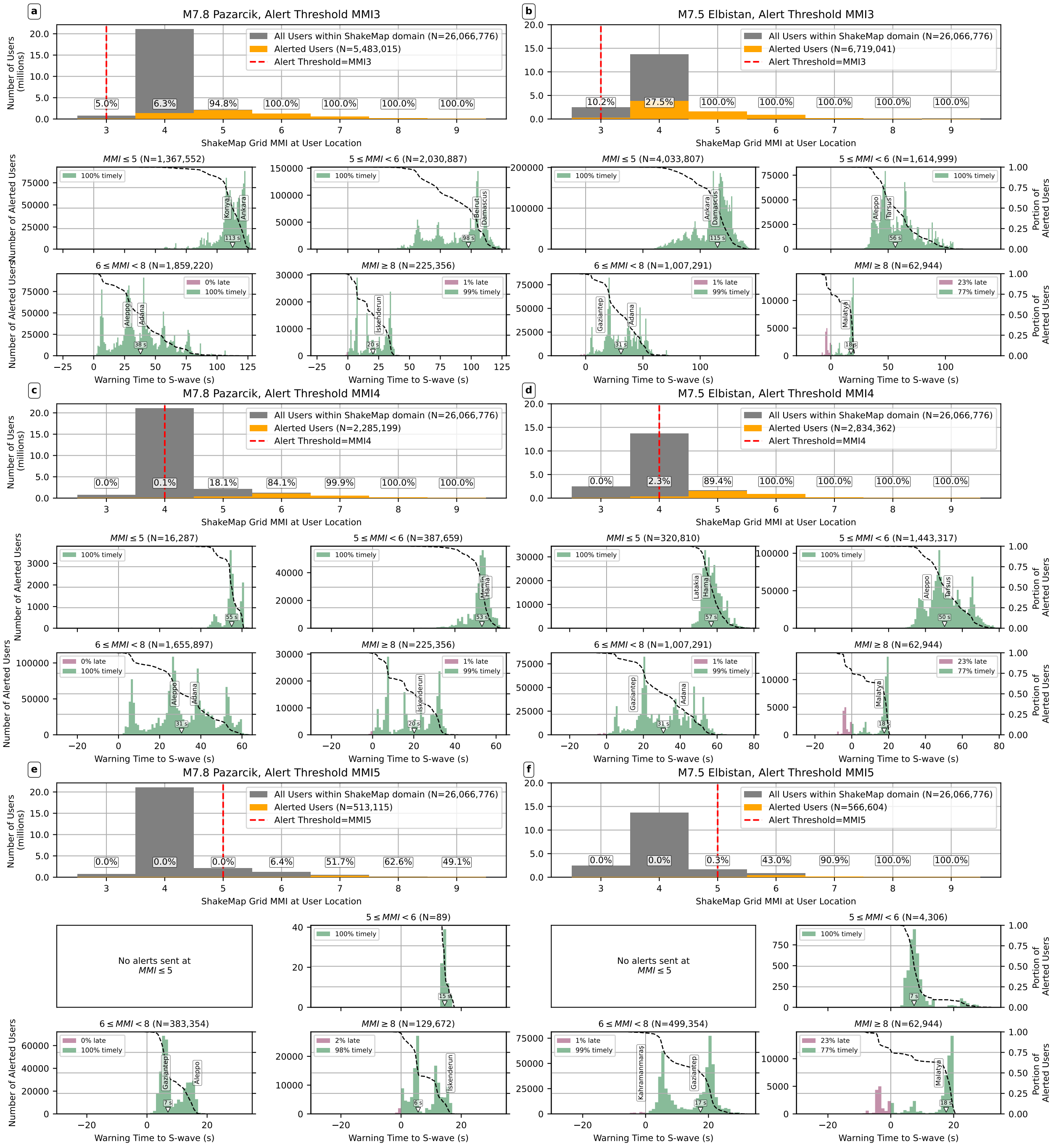

Figure S13: As in Figure 5 and 7 in the Main Text, but now plotting the alert quality and timeliness analysis for warning times calculated without the delivery latency. Left column (panels a, c, e) show results for the Pazarcık event, right column (panels b, d, f) for the Elbistan event. The warning time histograms use the exact same binning scheme used in Figures 5 and 7. Note how warning time peaks largely correspond to peaks in Figures 5 and 7 in the Main Text; these are driven by population density. We illustrate this by labeling the 2 most populous cities in each MMI bin, like in Figures 5 and 7 - note these are unchanged. However, note how the peaks here are much sharper. Including latency increases warning time variability and broadens peaks in the warning time distribution. Without latency, the estimate of the empirical complementary cumulative density function (ECCDF, which is 1 - ECDF) here is much steeper and step-like here compared to Figures 5 and 7.

Warning Time Analysis Considering MMI Exceedance Times

The main focus of this manuscript is to produce warning time estimates in the mass public alerting use case. We choose to do this by modeling warning times with respect to the arrival of the S-wave. However, many EEW studies define warning time with respect to the arrival time of ground-motion at a given level of intensity. In this section, we repeat the analysis of Figure 8 for the Elbistan earthquake (Figure S14). Figure S14 shows that over a large epicentral distance range, the arrival of the P-wave, approximates the arrival of MMI 3 ground-motion, while the arrival of the S-wave seems to approximate the arrival of MMI 5 shaking, which is also the onset of damage. As such, for the Elbistan earthquake, using the S-wave time as a proxy for the arrival of damaging ground shaking is a reasonable assumption.

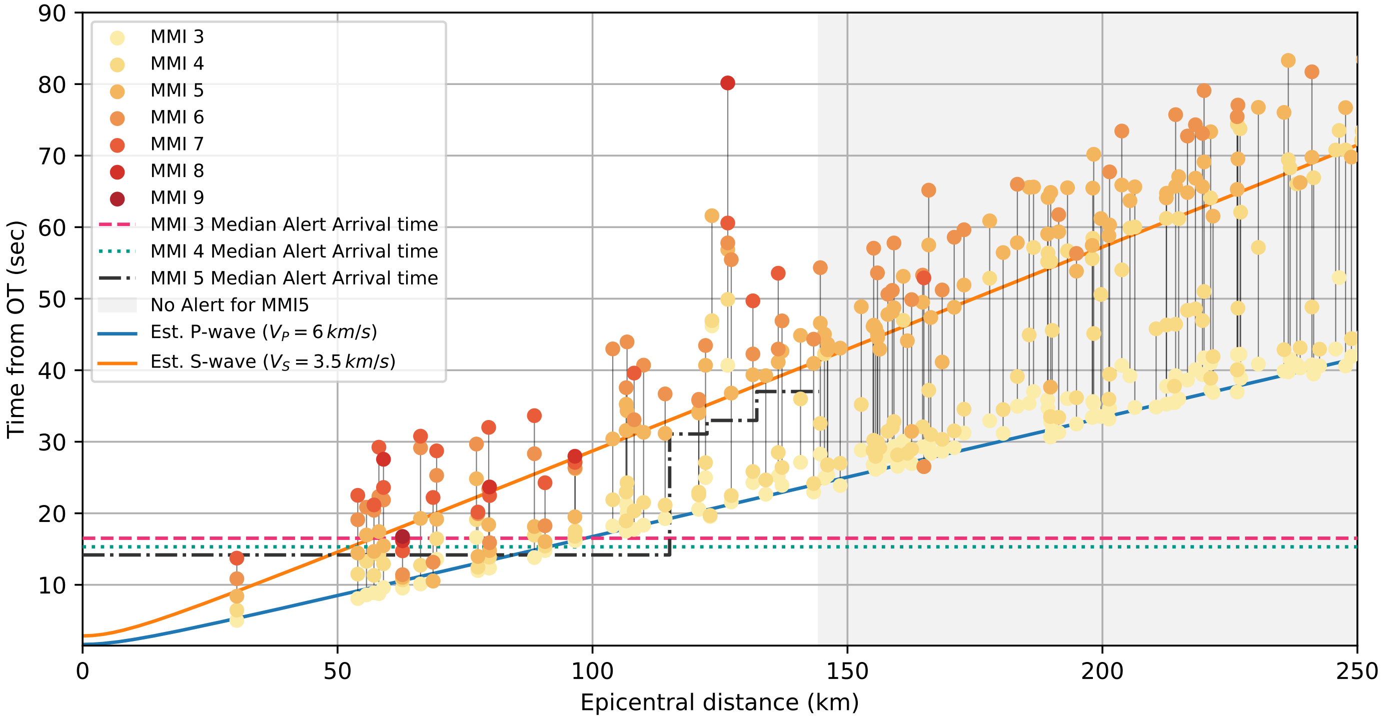

Figure S14: Analysis of MMI exceedance times with respect to alert arrival times for the $M7.5$ Elbistan earthquake. Follows the same structure as Figure 8a) in the Main Text.

As describe in the Main Text, while our focus is on mass public alerting targeting users distributed everywhere within our study domain, we also analyze warning times to different MMI levels on a per-station basis, leveraging the dense station coverage, especially in the Pazarcik event. We use these results to enable our comparison with results from replays of the Finder algorithm (see Main Text). For each station, we calculate the arrival time of different MMI levels, by using equally-weighted converted MMI from ground velocity and acceleration timeseries[24]. Given the timing of first arrival of different MMI levels, we calculate what the warning times would be with respect to the first exceedance of various MMI levels (i.e. the time between the alert arrival, accounting for time-evolutive alert issuance, and using the median alert delivery from our simulation, and the time at which the MMI level in question is first exceeded at a given station). We calculate these warning times under our three modeled alert thresholds. Results are given for the Pazarcik and Elbistan events in Figures S15 and S16, respectively. In general, we see that warning times are longer when calculated with respect to the first exceedance of higher MMI levels. Little difference is seen between the first and second columns, calculated using alert thresholds of MMI 3 and 4, respectively. Warning times are on average (compare medians, p_{50}, reported in the text box in each panel), much shorter for the simulation using MMI 5 alerting (third column).

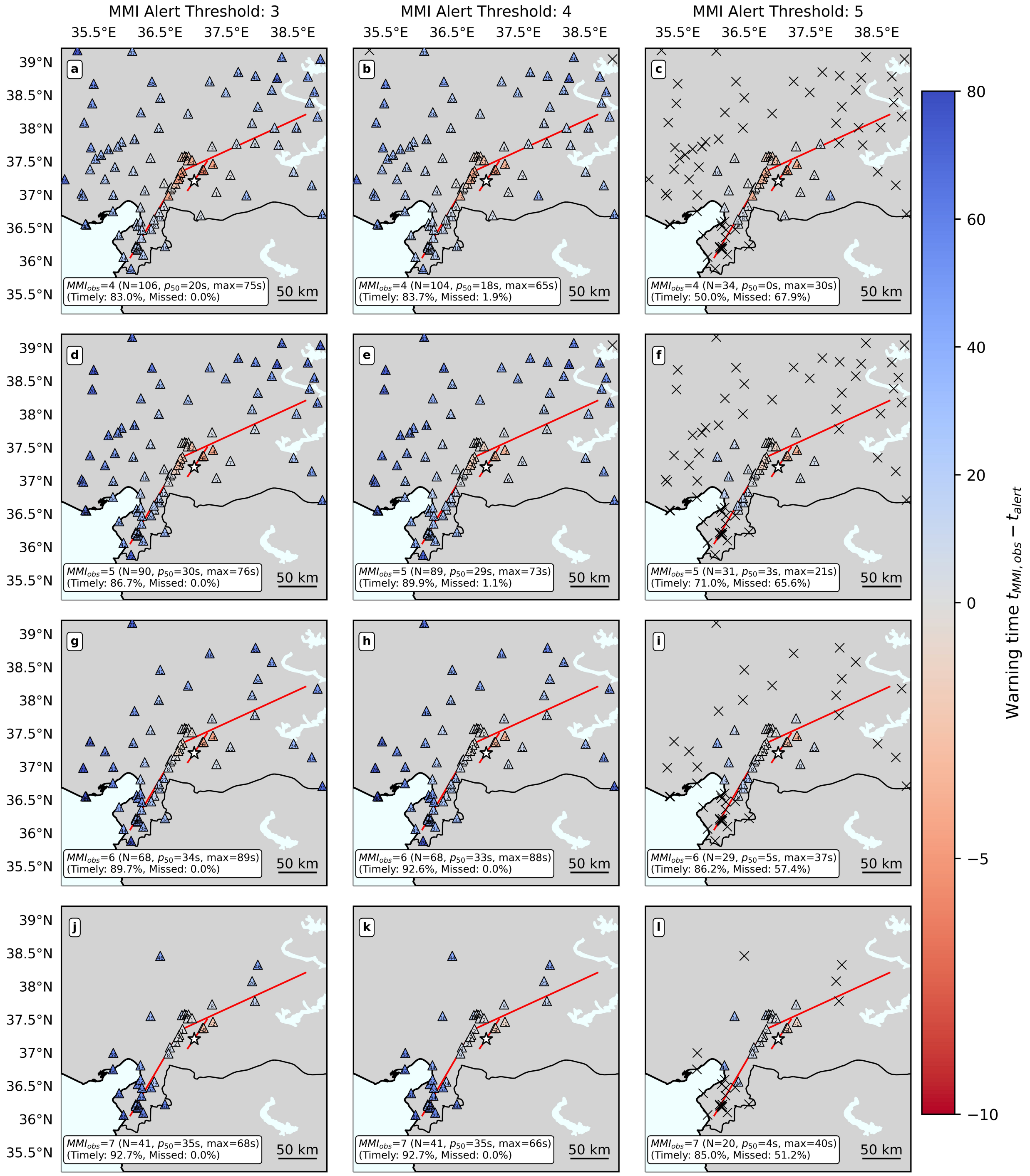

Figure S15: Warning time to different MMI levels for individual stations, under the three different alert thresholds for the Pazarcık event. Each column shows warning times for a a different alert threshold: left column (a, d, g, j), MMI 3; middle column (b, e, h, k), MMI 4; right column (c, f, i, l), MMI 5. Each row shows warning times to a different MMI level: First row (a-c), MMI 4, second row (d-f), MMI 5; third row (g-i), MMI 6; fourth row (j-l), MMI 7. We calculate warning time as the difference between the time at which the station first experiences a given MMI level ($t_{MMI,obs}$) and the time it is alerted (t_{alert}). t_{alert} includes latency and is calculated from interpolation using the method described in the Supplementary Text and Figure S12. We show the warning times with respect to the first exceedance of MMI 4, 5, 6, and 7, going down the rows. In each panel, the text box shows how many of the alerts sent were timely, as well as how many of the stations that experienced MMI_{obs} did not get alerted (missed alerts). We also show the median (p_{50}) and maximum warning time for all plotted stations. The alerted stations are colored and labeled by warning time. Stations that were missed (not alerted at all) are plotted as crosses.

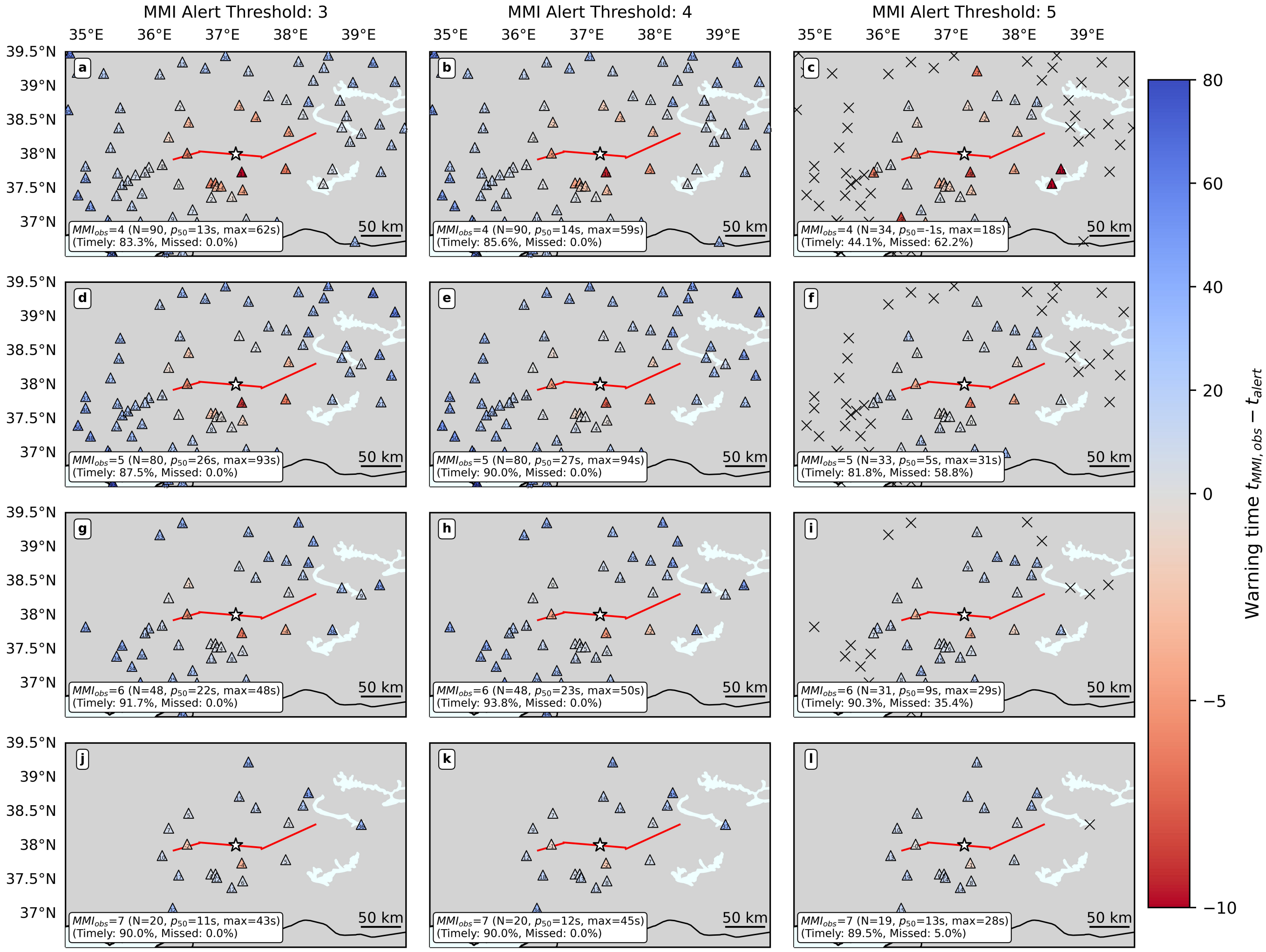

Figure S16: Same as Figure S16, but for the Elbistan event.

References

[1] Kohler, M. D. *et al.* Earthquake Early Warning ShakeAlert 2.0: Public Rollout. *Seismol. Res. Lett.* **91**, 1763–1775, DOI: 10.1785/0220190245 (2020).

[2] McGuire, J. J. *et al.* ShakeAlert® Version 3: Expected Performance in Large Earthquakes. *Bull. Seismol. Soc. Am.* **115**, 533–561, DOI: 10.1785/0120240189 (2025).

[3] Serdar Kuyuk, H. *et al.* Designing a Network-Based Earthquake Early Warning Algorithm for California: ElarmS-2. *Bull. Seismol. Soc. Am.* **104**, 162–173, DOI: 10.1785/0120130146 (2013).

[4] Chung, A. I., Henson, I. & Allen, R. M. Optimizing Earthquake Early Warning Performance: ElarmS-3. *Seismol. Res. Lett.* **90**, 727–743, DOI: 10.1785/0220180192 (2019).

[5] Chung, A. I. *et al.* ShakeAlert Earthquake Early Warning System Performance during the 2019 Ridgecrest Earthquake Sequence. *Bull. Seismol. Soc. Am.* **110**, 1904–1923, DOI: 10.1785/0120200032 (2020).

[6] Williamson, A., Lux, A. & Allen, R. Improving Out of Network Earthquake Locations Using Prior Seismicity for Use in Earthquake Early Warning. *Bull. Seismol. Soc. Am.* DOI: 10.1785/0120220159 (2023).

[7] Lux, A. I. *et al.* Status and Performance of the ShakeAlert Earthquake Early Warning System: 2019–2023. *Bull. Seismol. Soc. Am.* DOI: 10.1785/0120230259 (2024).

[8] Kuyuk, H. S. & Allen, R. M. A global approach to provide magnitude estimates for earthquake early warning alerts. *Geophys. Res. Lett.* **40**, 6329–6333, DOI: 10.1002/2013GL058580 (2013).

[9] Melgar, D. *et al.* Sub- and super-shear ruptures during the 2023 Mw 7.8 and Mw 7.6 earthquake doublet in SE Türkiye. *Seismica* **2**, DOI: 10.26443/seismica.v2i3.387 (2023). Number: 3.

[10] Goldberg, D. E. *et al.* Rapid Characterization of the February 2023 Kahramanmaraş, Türkiye, Earthquake Sequence. *The Seism. Rec.* **3**, 156–167, DOI: 10.1785/0320230009 (2023).

[11] Jia, Z. *et al.* The complex dynamics of the 2023 Kahramanmaraş, Turkey, Mw 7.8-7.7 earthquake doublet. *Science* **381**, 985–990, DOI: 10.1126/science.adi0685 (2023). Publisher: American Association for the Advancement of Science.

[12] Savage, J. C. Radiation from supersonic faulting*. *Bull. Seismol. Soc. Am.* **61**, 1009–1012, DOI: 10.1785/BSSA0610041009 (1971).

[13] Nagasaka, Y. & Nozu, A. Kinematic Source Properties of the 2023 Mw 7.7 Türkiye Earthquake Inferred from Near-Fault Strong Ground Motions. *Seismol. Res. Lett.* **96**, 19–34, DOI: 10.1785/0220240156 (2024).

[14] Heath, D. C., Wald, D. J., Worden, C. B., Thompson, E. M. & Smoczyk, G. M. A global hybrid VS30 map with a topographic slope–based default and regional map insets. *Earthq. Spectra* **36**, 1570–1584, DOI: 10.1177/8755293020911137 (2020).

[15] Patel, S. C. & Allen, R. M. The MyShake App: User Experience of Early Warning Delivery and Earthquake Shaking. *Seismol. Res. Lett.* **93**, 3324–3336, DOI: 10.1785/0220220062 (2022).

[16] Marcou, S. *et al.* Insights into ShakeAlert Earthquake Early Warning Alert Delivery by the MyShake Platform from Rapid Post-Event Analyses. *Seismol. Res. Lett. (in press)* (2025).

[17] Thakoor, K., Andrews, J., Hauksson, E. & Heaton, T. From Earthquake Source Parameters to Ground-Motion Warnings near You: The ShakeAlert Earthquake Information to Ground-Motion (eqInfo2GM) Method. *Seismol. Res. Lett.* **90**, 1243–1257, DOI: 10.1785/0220180245 (2019).

[18] Hoffman, M. D. & Gelman, A. The No-U-Turn Sampler: Adaptively Setting Path Lengths in Hamiltonian Monte Carlo, DOI: 10.48550/arXiv.1111.4246 (2011). ArXiv:1111.4246 [stat].

[19] Vehtari, A., Gelman, A. & Gabry, J. Practical Bayesian model evaluation using leave-one-out cross-validation and WAIC. *Stat. Comput.* **27**, 1413–1432, DOI: 10.1007/s11222-016-9696-4 (2017).

[20] Watanabe, S. Asymptotic Equivalence of Bayes Cross Validation and Widely Applicable Information Criterion in Singular Learning Theory. *J. Mach. Learn. Res.* **11**, 3571–3594 (2010).

[21] WorldPop (www.worldpop.org - School of Geography and Environmental Science, University of Southampton, Department of Geography and Geosciences, University of Louisville; Departement de Geographie, Universite de Namur) & Center for International Earth Science Information Network (CIESIN), Columbia University. Global High Resolution Population Denominators Project - Funded by The Bill and Melinda Gates Foundation (OPP1134076)., DOI: https://hub.worldpop.org/doi/10.5258/SOTON/WP00671 (2018).

[22] Kong, Q., Martin-Short, R. & Allen, R. M. Toward Global Earthquake Early Warning with the MyShake Smartphone Seismic Network, Part 2: Understanding MyShake Performance around the World. *Seismol. Res. Lett.* **91**, 2218–2233, DOI: 10.1785/0220190178 (2020).

[23] Kong, Q., Martin-Short, R. & Allen, R. M. Toward Global Earthquake Early Warning with the MyShake Smartphone Seismic Network, Part 1: Simulation Platform and Detection Algorithm. *Seismol. Res. Lett.* **91**, 2206–2217, DOI: 10.1785/0220190177 (2020).

[24] Worden, C. B., Gerstenberger, M. C., Rhoades, D. A. & Wald, D. J. Probabilistic Relationships between Ground-Motion Parameters and Modified Mercalli Intensity in California. *Bull. Seismol. Soc. Am.* **102**, 204–221, DOI: 10.1785/0120110156 (2012).
